# Natural resources used in the traditional medicine of the Marinaú community, Caxiuanã forest, Brazil

**DOI:** 10.3389/fphar.2024.1443360

**Published:** 2024-10-09

**Authors:** Paula Maria Correa de Oliveira, João Paulo Barreto Sousa, Lorena C. Albernaz, Laila Salmen Espindola, Márlia Coelho-Ferreira

**Affiliations:** ^1^ Postgraduate Program in Biodiversity and Biotechnology, Universidade Federal do Pará, Programa de Pós-Graduação em Biodiversidade e Biotecnologia, Belém, PA, Brazil; ^2^ Laboratory of Pharmacognosy, Pharmacy Department, Universidade de Brasília, Campus Universitário Darcy Ribeiro, Brasília, DF, Brazil; ^3^ Laboratory of Ethnobotany, Botany Department, Museu Paraense Emílio Goeldi, Belém, Brazil

**Keywords:** ethnobothany, ethnozoology, Amazonian populations, ethnomedicine, Amazonian sustainable use conservation units

## Abstract

**Background:**

The diversity of Conservation Units in Brazil, ranging from Strict Protection areas like National Parks to Sustainable Use areas such as National Forests, supports the coexistence of human populations with extensive traditional knowledge of local natural resources. This traditional and local knowledge plays a crucial role in their subsistence and has significant potential to contribute to bioprospecting initiatives, as well as to enhance research and strategies for biological conservation. The São Sebastião de Marinaú community, situated within the Caxiuanã National Forest, offers valuable opportunities for ethnobiological studies.

**Methods:**

The field study was carried out with 48 informants from the São Sebastião de Marinaú Community, Caxiuanã National Forest (CNF), Brazil, a Sustainable Use Conservation Unit. It involved participant observation, semi-structured interviews, and guided tours. Indices of Use Value (UV), Fidelity Level (FL), and Consensus Factor (ICF) indicated plant species with therapeutic potential.

**Results:**

A total of 944 uses associated with 154 plants and 21 animals were reported. Statistical tests pointed out that factors such as gender and age are not determinants in the richness of known plants among the residents. The plants are distributed among 59 botanical families and 126 genera. They predominantly use species native to Brazil (69%), among which 47 are endemic to the Amazon. The medicines are prepared mainly by decoction of leaves and bark. According to UV, veronica (*Dalbergia monetaria* L.f.) was the most important. The animals used are all vertebrates, and paca (*Cuniculus paca* Linnaeus, 1766,) was the most cited. Bile and lard are the parts most used in the recipes. They mentioned 116 diseases, especially those of the digestive system. The ceruzeiro (*Allantoma lineata* (Mart. ex O.Berg) Miers) had a high consensus of local use, and no additional studies on this species exist.

**Conclusion:**

This study underscores the vital role of traditional communities in sustainable conservation units, as their involvement is crucial for preserving plant and animal species essential to local traditional medicine. Such research also promotes the recognition of non-timber products as valuable raw materials with potential applications in the chemical and pharmaceutical industries. Additionally, mapping the occurrence and use of species in vulnerable conditions aids in developing effective conservation strategies for these resources.

## 1 Introduction

Brazil’s legal Amazon covers approximately five million km^2^, representing about 60% of the country’s total land area (*IBGE atualiza Mapa da Amazônia Legal | Agência de Notícias*, 2020). The National System of Nature Conservation Units, established in July 2000 by Law No. 9,985, facilitates the organization of these areas at the federal, state, and municipal levels. In Brazil, there are several types of Conservation Units, including Full protection units such as National parks or Sustainable Use Units such as National forests. In this regard, conservation units are among the best strategies for protecting natural attributes and heritage. In these areas, the plant, animal, and microorganism species and the ecological processes that govern the ecosystems are conserved, guaranteeing the maintenance of biodiversity. The first Sustainable Use Conservation Unit in the Brazilian Amazon was the Caxiuanã National Forest (CNF), created in November 1961 (official decree No. 239), with 3,179.50 km^2^ of sustainable area, representing 0.05% of the area of this biome (Miranda et al., 2015).

The CNF encompasses 15 surrounding communities located in the state of Pará, Brazil. These communities are home to riverside populations with an Amazonian identity: descendants of Black, Indigenous, and White peoples. These populations have accumulated knowledge on using valuable natural resources for their livelihoods and play a vital role in biodiversity conservation (Miranda et al., 2015). Ethnobotanical and ethnopharmacological research carried out in the Amazon has documented the use of plant resources as either the primary or sole option in healthcare. These studies reveal shifts or losses in traditional knowledge (Fonseca-Cepeda et al., 2019), emphasize the importance of understanding the emic perspective on therapeutic practices (Pagani et al., 2017) and identify plants or animals with significant potential for bioprospecting research (Bieski et al., 2015; Ribeiro et al., 2017; Santos et al., 2012).

In addition, ethnobiological studies play a crucial role in biocultural conservation, integrating traditional and scientific knowledge to preserve biodiversity and associated cultures. In the Amazon, for example, research into medicinal plants reveals the vast expertise of riverine and indigenous populations in using plant species for therapeutic purposes, contributing to the appreciation and conservation of forest ecosystems and local cultural practices. Penedo et al. (2023) highlight the complexity of traditional knowledge associated with indigenous populations when compared to other populations, revealing the need for studies that document traditional knowledge to create new conservation strategies and public policies. Pedrollo et al. (2016) conducted a survey among riverine populations in which it was observed that native plants are fundamental to local subsistence and that traditional practices should be considered when making decisions regarding local public conservation policies. Considering that medicinal plants are a resource of global use for populations all over the world and that this can generate constant pressure on existing resources, the documentation of traditional knowledge is essential for the practice of strategies proposed by Mir et al. (2021) that includes the *in situ* conservation by establishing preservation areas and cultivation, as well as projects including botanical gardens and gene banks in *ex-situ* conservation.

Since 1990, the Paraense Emílio Goeldi Museum has established the Ferreira Penna Scientific Station in the CNF. It serves as an essential hub to support studies on biodiversity conservation and socio-environmental issues, facilitating research and innovation, conducting science education activities, and promoting environmental education (Lisboa et al., 2013). The studies have already reported more than 2,400 species of plants distributed in all environments, including dense ombrophilous dryland forests, floodplains, alluvial dense ombrophilous lowland, campinarana, and cerrado regions. For the vast majority of these species, local knowledge has been associated with scientific experiments that have revealed various biological activities. These include antifungal, antibacterial (Júnior et al., 2023), leishmanicidal (Odonne et al., 2011), larvicidal (Correa de Oliveira et al., 2022), antiplasmodic, antimalarial (Viana dos Santos et al., 2024), and antioxidative activities (Ferreira et al., 2022). Many of these activities could be related to the presence of secondary metabolites such as derivatives of isoflavonoids, saponins, phenylpropanoids, and benzophenones.

Currently, in the CNF, there are human influences across all its regions, with a notable concentration in the western portion next to the municipalities of Porto de Moz, Gurupá, and Senador José Porfírio. Less than 20% of the CNF’s area has been covered by the research conducted thus far (Lisboa et al., 2013). Among its 15 communities, only one - coincidentally named Caxiuanâ Community - has been the focus of an ethnobotanical study, specifically emphasizing the use of forest plant species in local homemade medicine (Rocha et al., 2013). Thus, it is undeniable how important it is to develop new research that documents the local vegetation, customs, and associated knowledge of this CNF, considering the long distances between communities, which promotes the development of specific knowledge. Additionally, this study did not include quantitative analyses showing lists of important local species or reports on the use of other natural resources in healthcare.

In 2018, we initiated a groundbreaking study involving the São Sebastião de Marinaú riverside community in the CNF. The initial phase of this study focused on bioprospecting for new larvicides targeting the *Aedes aegypti* vector, utilizing ethnobiological knowledge from the community regarding repellent plants (Oliveira et al., 2022). However, the diverse uses of plants within this community warrant a broader ethnobiological research. The traditional knowledge held by riverside communities residing in sustainable use conservation areas could serve as a foundation for new bioprospecting endeavors or contribute to the advancement of biological conservation studies and strategies. Therefore, this research sought to conduct a survey of the natural resources used in the community of Marinaú in order to understand their importance in local healthcare, their preparation, application methods, and to identify the useful resources for chemical and pharmacological studies, as well as for conservation.

## 2 Material and methods

### 2.1 Ethical procedures

The project was approved by the Research Ethics Committee (CEP) of the Federal University of Pará (UFPA/ICS no 3.965.175) and registered in the National System for the Management of Genetic Resources and Associated Traditional Knowledge (SisGen n° AE259B4). All plant collections were performed with authorization from the Biodiversity Authorization and Information System (SISBIO, Process n° 68325).

### 2.2 Investigation site and social aspects

**FIGURE 1:**
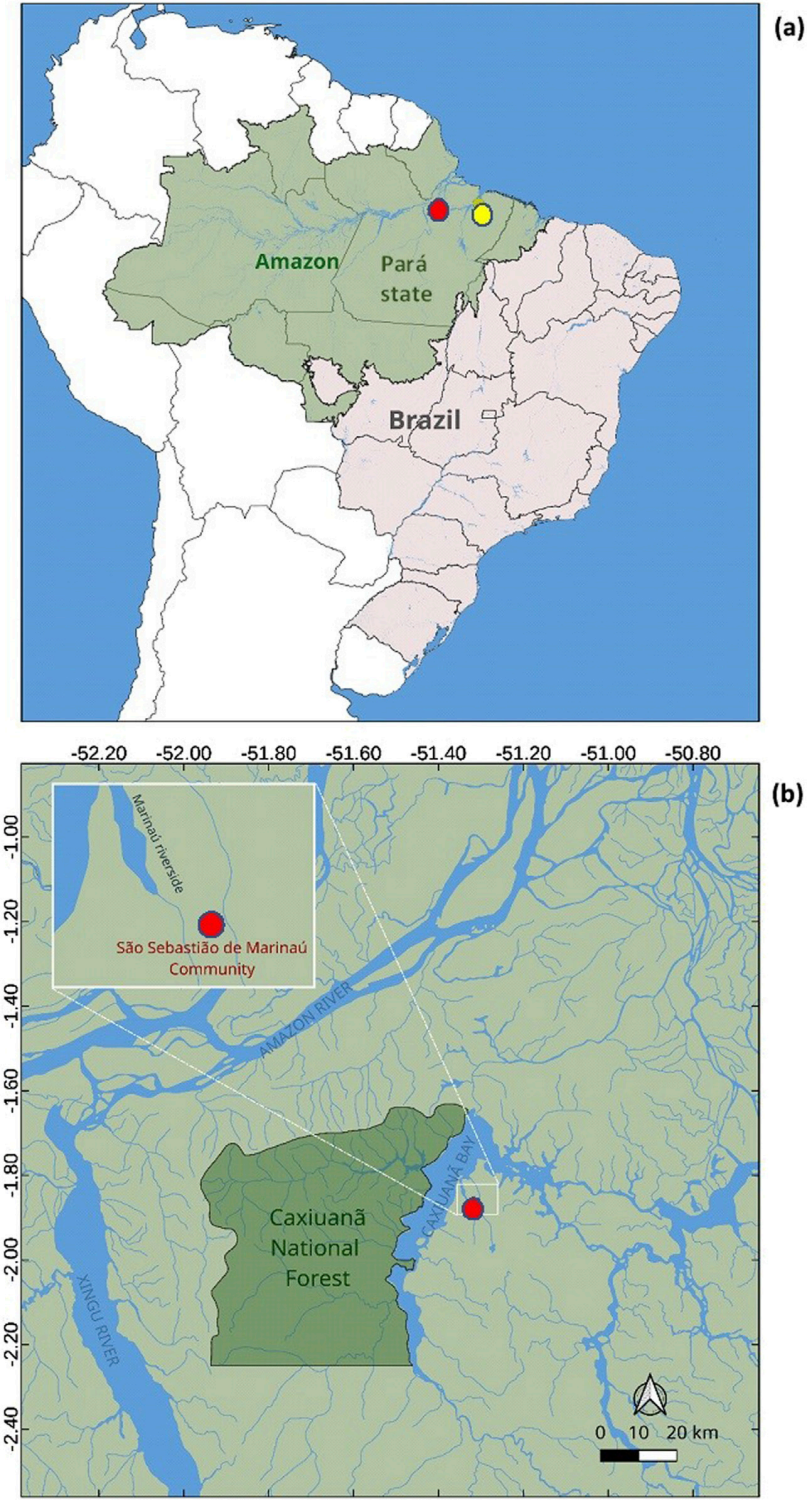
**(A)** Geographic localization of the São Sebastião de Marinaú Community (red) 300 km from Belém city (yellow). **(B)** Caxiuanã National Forest (01°42′30″S and 51°31′45″W), Brazil, where the traditional knowledge and associated samples were collected.

The CNF is located between the municipalities of Portel and Melgaço, in northeastern Pará State, northern Brazil, with coordinates 01°42′30″S, 51°31′45″W and an elevation of 19–47 m above sea level (Figure 1A). This CNF is located more precisely in the Marajó archipelago at the interfluve of the Xingu and Amazon rivers on the banks of the Anapu river, which is 470 km in length. The distances and access to the forest involve the routes between the city of Belém and the municipality of Breves: 126 nautical miles - 12 h by boat; 1- hour flight. Afterwards, from Breves to CNF: 64.38 nautical miles – 8 h by boat, in a 16.5 hp motor boat. Other routes include Portel to CNF: 42.84 nautical miles – 5 h by boat, in a 16.5 hp motor boat or Melgaço to CNF: 49.45 nautical miles – 6 h by boat, in a 16.5 hp motor boat. Of these, 85% are dense ombrophilous floodplain forests, and the remaining area is alluvial dense ombrophilous lowland (igapó) forests. As such, its access is predominantly by the river, approximately 328 km from the capital Belém. The average annual temperature is 2 + 5.7°C ± 0.8°C, and the relative humidity is nearly 80%. According to the Köppen classification, the climate is tropical “Am,” with annual precipitation ranging from 2,500 to 3,000 mm (Lisboa, 2012).

The traditional medical knowledge field study was conducted in the São Sebastião de Marinaú Community between June 2018 and May 2019, one of 14 communities located in the CNF (Figure 1B). This community has approximately 150 inhabitants, corresponding to 30 families. In the field study, 48 individuals were interviewed (32% of residents) including 32 adults and 16 adolescents. The adolescents were literate and enrolled in the local school. The adults comprised 18 women and 14 men, ranging in age from 20 to 68 years old. They have lived in this community since its was established approximately 40 years ago.

For survival, local agricultural activities are restricted to growing and processing cassava (*Manihot esculenta* L.). They also raise domestic animals, such as chickens and ducks, as well as wild animals like as pacas, monkeys, and sloths, which can be used for consumption. The community has an elementary school, a Catholic church, and a community house, where they gather for festivities and decision-making. There is no basic sanitation or assistance from health centers. The families live in wooden houses with backyards and farmland areas surrounded by vegetation cover. There are a few “flour houses” used collectively by local families for processing cassava into flour. Table 1 contains information taking into account the informants’ social aspects.

**TABLE 1:** Social aspects of residents of the São Sebastião de Marináú Community, CNF, Brazilian Amazon.

Topics	Proportion	%
Gender		
Male	19	39.58
Female	29	60.42
Age of respondents (years)		
15–19 (adolescent)	16	33.33
20–30	7	14.58
31–40	8	16.67
41–50	6	12.50
51–68	11	22.92
Educational status		
None	16	33.33
Primary	32	66.67
Occupation		
Student	16	33.33
Small farmers	16	33.33
Housewife	8	16.67
Extractivist	7	14.58
Accoucheuse	1	2.08
Religion		
Protestantism	3	6.25
Catholic	40	83.33
None	5	10.42

### 2.3 Informant selection and data collection

The participants selected were adults and adolescents. Adults comprised at least one representative from each of the 30 households in the study area, including the head of the household, aged 18 years or older. Adolescents were aged 14–17 years, literate, and enrolled in the local school.

**FIGURE 2:**
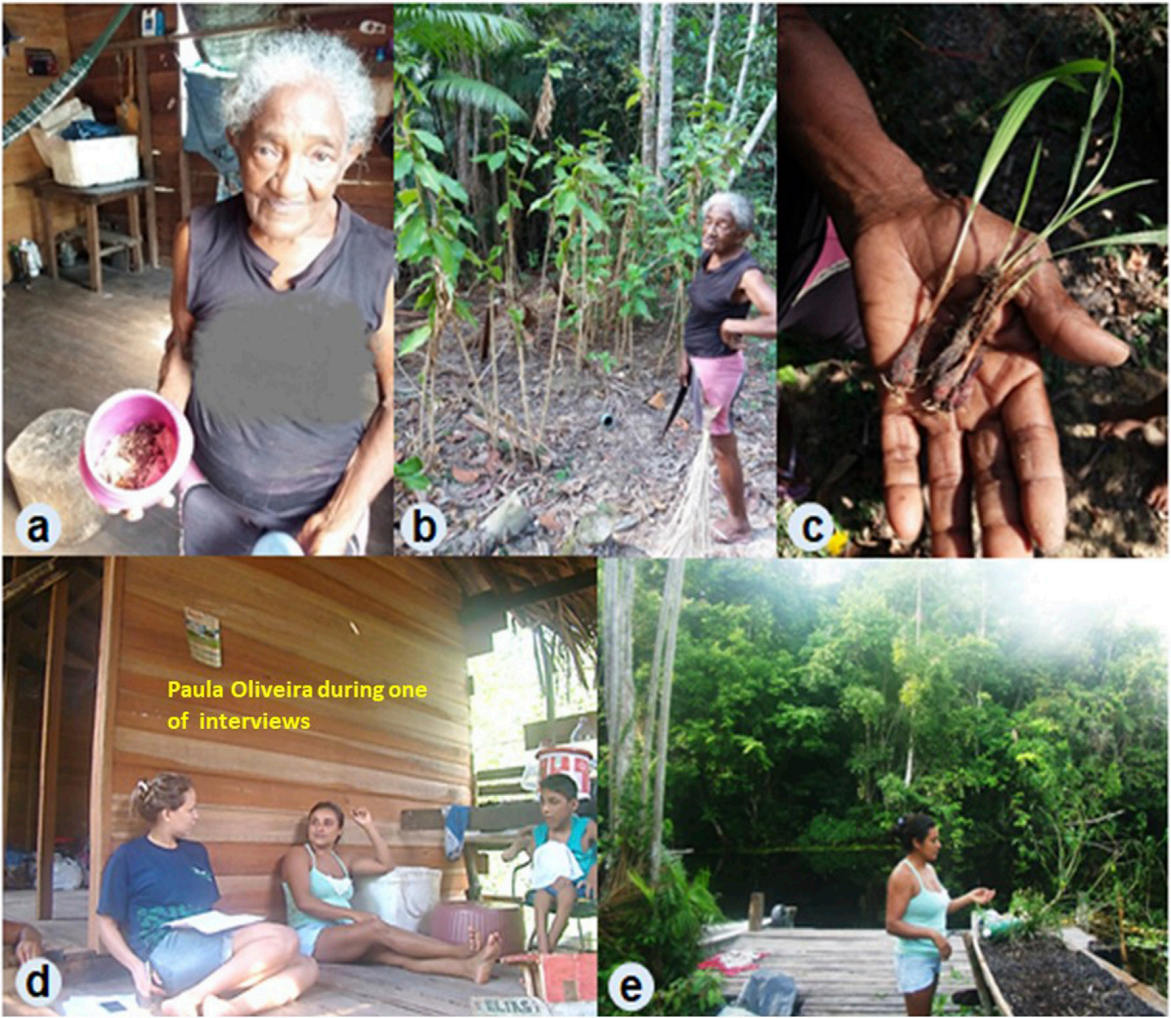
Application of semi-structured interviews followed by walks in residential backyards and riverines houses from São Sebastião de Marináú Community. **(A–C)** Residence of Mrs. Helene da Silva, 68 years old. **(D, E)** Residence of Mrs. Vanderleia de Araújo, 39 years old.

The traditional medical knowledge of the adults was documented during the visit to their homes (Figure 2). The adults’ data were obtained through semi-structured interviews and free listing. The semi-structured interviews were based on a flexible script containing a list of topics to be addressed, allowing us to characterize the sociocultural profile, medicinal recipes, diseases, and places where the species indicated for treatment are found. The free-listing technique consisted of asking informants to list the medicinal plants or animals they knew and/or used.

The adolescents’ knowledge was obtained during extracurricular activities held during breaks or after school. Initially, we offered workshops to the students to help them correctly identify the various plant organs. At another time, the teenagers were asked to mention the plants they knew and/or used, along with their therapeutic indications, the parts used, and how they were prepared, as well as drawings of the plants. We then visited the school’s medicinal garden and its surroundings with all the teenagers so that the plants they had mentioned could be properly identified and collected. The drawings and plant lists were used to compare with the information from the adults. This technique supported the collection and identification, especially of plants.

### 2.4 Species sampled

During the field research, 252 vascular plant specimens were collected with the informants during guided tours (walking in the woods). These samples (3-5 aliquots per species) were herborized, identified, and deposited in the Herbarium MG of the Emílio Goeldi Museum of Pará. Infertile samples were incorporated into the Ethnobotanical Collection of the same institution. The botanical classification system adopted was the *Angiosperm Phylogeny Group* - APG IV 2016 (The Angiosperm Phylogeny Group et al., 2016). The botanical nomenclature update and the geographic origin of the species were verified using the Plants of the World Online (POWO) database (https://powo.science.kew.org/). Most ethnospecies mentioned in the interviews were collected and identified at the species level. The minority were classified in terms of genera, and botanical family or just recorded by name and traditional use. The latter were not taken into account in the ethnobotanical indices analyses. The identification of the animals mentioned in the interviews and their organs used in therapeutic preparations was based on the morphological descriptions given by the informants. These data were compared with those found in faunal inventories already conducted in this Sustainable Use Conservation Unit to determine the species used (Lisboa et al., 2013; Lisboa, 2009 , 2012).

### 2.5 Data analysis

Statistical tests were performed based on the number of ethnospecies cited by each informant, or all the use-reports that is a citation of a plant taxon (or plant ethnospecies) by one participant for a particular use-category. The variables considered in the analyses were the gender and age of the informants. The analysis of variance (ANOVA) evaluated the relationship between the number of ethnospecies mentioned by each informant and their age, while the t-test was used to verify if there was a significant difference in knowledge between gender groups. The chi-square test was utilized to identify differences in use-reports numbers between women and man. R software (Chambers, 1998) was used for all these tests.

To analyze the level of fidelity regarding the use of a resource and its relative importance, we used the Fidelity Level (FL) and Use Value (UV). The Fidelity Level (FL) was used to evaluate the species most frequently used by riverside dwellers for each mentioned disease or symptom. This index can be estimated from the following equation: FL (%) = (NP/N) x 100, where, NP is the number of people who cited the species for a main therapeutic use and N is the total number of people who cited the species for any use. An FL value equal to 100% means that all participants use the species for a therapeutic application, while lower values mean that the species is used for different purposes (Friedman et al., 1986).

Use Value (UV) is a quantitative index that expresses the therapeutic importance of each species, being sensitive to the number of uses attributed to the species. It is calculated by the following equation: UV = Σ^Ui/N^, where UV is the Use Value of a species, Ui is the use-reports for each plant species, and N is the total number of respondents. The UV parameter helps to determine which plant is most often used for specific purposes (Logan and Michael, 1986).

Reported symptoms and illnesses were grouped according to the International Classification of Primary Care (ICPC-2) (Miller et al., 2009). This classification clusters the diseases based on body systems and psychological and social problems. The ICPC considers the patient’s perceptions to construct the categories (Staub et al., 2015). In order to analyze which body systems had the greatest agreement among the informants regarding the use of plants and animals, the Informants Consensus Factor (ICF) was used, obtained by the equation: ICF = (Nur–Nt)/(Nur – 1). Where, Nur refers to the number of use-reports for a particular resource and Nt refers to the number of taxa used for a particular resource by all informants. The product of this factor ranges from 0 to 1. A high value (close to 1.0) indicates that relatively few taxa are used by a large proportion of the informants. A low value indicates that the informants disagree on the taxa to be used in the treatment within a category of illness (Logan and Michael, 1986).

## 3 Results and discussion

### 3.1 Knowledge of natural resources in Marinaú's traditional medicine

All participants declared that they know and use medicinal plants or animal parts as a primary therapeutic alternative. In situations where they lack knowledge, they seek help from local experts. When local natural resources are ineffective in treating a given disease or in urgent cases, they travel to the Ferreira Penna Scientific Station, which is about 4 hours away by river and periodically has an infirmary and riverboat to send them to specialized care in nearby cities.

A total of 944 uses associated with 175 natural resources (154 plants and 21 animals) were reported. Most of these uses were mentioned by women (629 reported uses). Women represented 60.42% of the participants. Men identified 130 ethnospecies, while women identified 147. Regarding the acquisition of knowledge about the use of medicinal plants, all informants acquired it from their relatives (parents, grandparents, and other relatives), either during daily living or during agro-extractive activities. The number resources here were relatively high compared to those observed in other studies conducted in riverside communities in the Amazon (Sarquis et al., 2019; Rocha et al., 2013; Santos et al., 2012).

Statistical tests pointed out that factors such as gender and age are not determinants in the richness of known plants among the residents (tgender test: *t* = - 0.80415, *p* > 0.05). On the other hand, there was a significant difference in the number of uses reported between men and women, indicating that women have a broader knowledge of the uses associated with medicinal resources (chisquare test *p* > 0.05). In this community, it was observed that adolescents frequently mention herbaceous and arboreal plants from which they also consume the fruit, such as *Anacardium occidentale* L., *Mangifera indica* L., *Annona mucosa* Jacq., *Euterpe oleracea* Mart., and others. We also noticed some plants mentioned only by the adolescents, such as *Physalis angulata* L. According to them, this species was brought by one of their teachers to the school’s medicinal garden, on the grounds that the plant could be used to treat malaria. Regarding to the uses and preparation methods of many species, the adult informants showed more knowledge than the youngsters.

Even if slowly, there is a dynamic in the construction of this knowledge. Adolescents perceive and use plants in a way that is still very similar to that of their families, but it would not be strange to notice changes in these plant repertoires over the years, considering the constant environmental changes that can occur in the area, as well as the circulation of new information and even plant species. The study carried out by Sousa et al. (2022) showed how the repertoire and knowledge about plants can be dynamic over time in a given community.

### 3.2 Medicinal plants used in the São Sebastião de Marinaú community

The São Sebastião de Marinaú community’s river dwellers reported using 154 ethnospecies to treat different diseases, 136 of which were identified at the species level, 10 at the genus level, and six that could not be collected and determined (Supplementary Table 1). The richness of plants recorded here is relatively high when compared to those observed in other studies in Amazonian riverine communities, whose sample universe was larger (Rocha et al., 2013; Pedrollo et al., 2016; Sarquis et al., 2019). The amount of species is relatively high. The species in this survey are distributed in 59 botanical families and 126 genera. Fabaceae (17 species), Lamiaceae (11 species), and Asteraceae (8 species) were the most representative botanical families. In floristic inventories already conducted in Caxiuanã, Fabaceae are among the most common and abundant families (Fernandes et al., 2020).

They predominantly use species native to Brazil (69%), among which 47 are endemic to the Amazon. Regarding habit, 47% of the species are arboreal, followed by herbaceous (38%), shrubs (8%), and liana (7%). The expressive use of arboreal and native plants is common among riverine populations in the Amazon (Sarquis et al., 2019; Pedrollo et al., 2016; Ribeiro et al., 2017; Rocha et al., 2013), who take advantage of them either in traditional medicine, food, income generation or even to obtain wood for building houses and work tools. Some of these species are endangered or vulnerable, such as *Bertholletia excelsa* Bonpl., *Virola surinamensis* (Rol. ex Rottb.) Warb., and *Vouacapoua americana* Aubl., *Manilkara elata* (Allemão ex Miq.). Previous research shows that these plants occur abundantly in this forest (Ferreira et al., 2005; Maciel, 2015), suggesting the contribution of this Sustainable Use Conservation Unit to their conservation. However, in recent years, concessions have been made for logging in the area (Governo Federal do Brasil, 2022). Their targets are species of therapeutic importance to local communities, such as *M. elata* (Allemão ex Miq.) Monach, *Hymenaea courbaril* L., and *Dipteryx odorata* (Aubl.) Forsyth f. Lisboa et al. (2013) have already alerted that even though they are legal practices, they contribute to environmental degradation. These authors emphasize the forest potential of CNF for the development of new possibilities of use by taking advantage of non-timber products, considering the local traditional knowledge.

**FIGURE 3:**
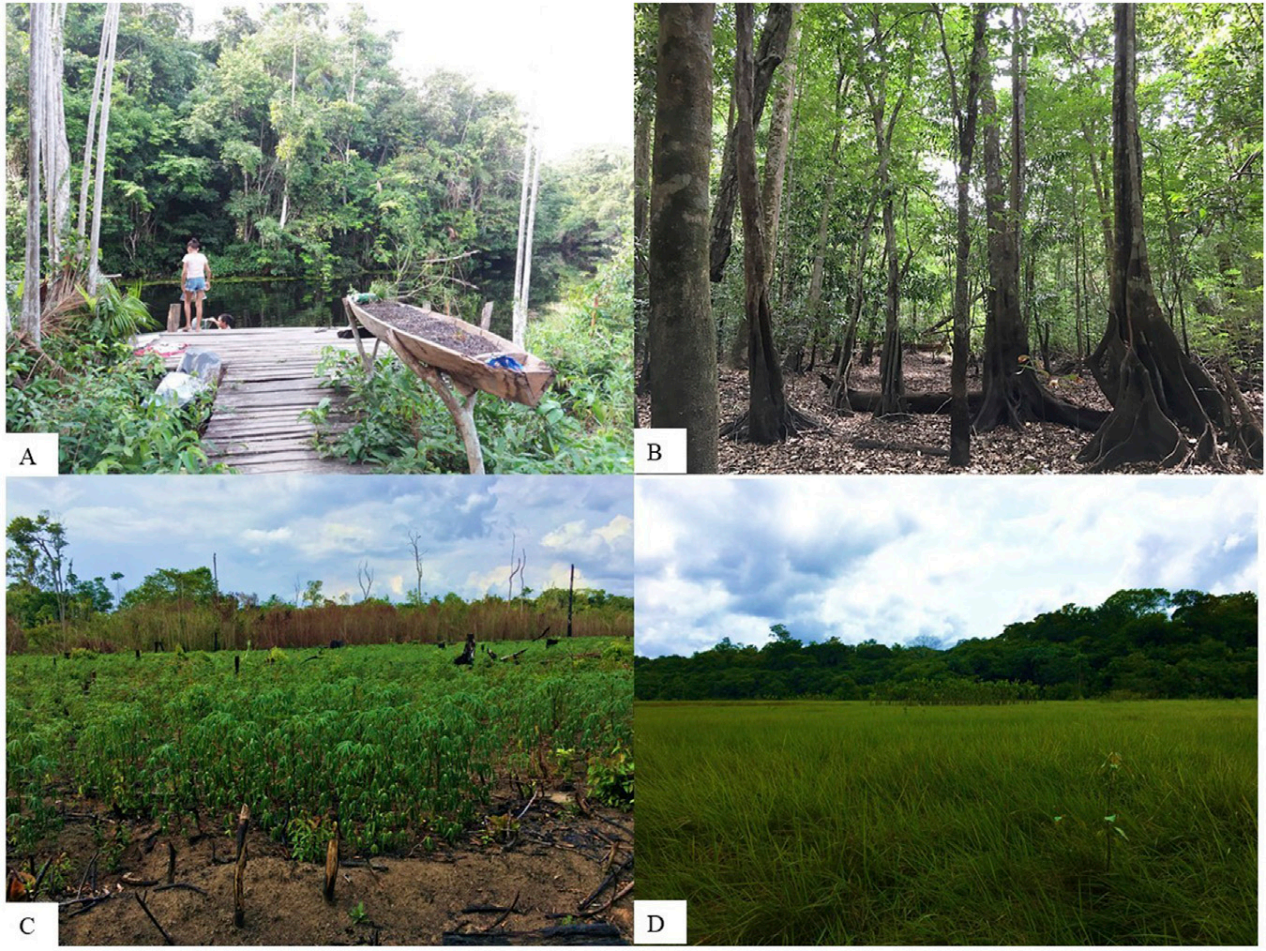
Environments managed by residents of the São Sebastião de Marináú Community, Flona de Caxiuanã, Pará, Brazilian Amazon. **(A)** Jiraus in backyard areas; **(B)** terra firme forest; **(C)** Clearing area; **(D)** Campinarana.

The main environments where the plants are sought are the backyards and the terra firme forest, from which the residents obtain parts of herbs and trees (Figure 3). For them, backyards are the areas surrounding the houses, where species such as *Lippia alba* (Mill.) N.E.Br. ex P. Wilson, *Centratherum punctatum* Cass., *Aeollanthus suaveolens* Mart. ex Spreng. and *Stachytarpheta cayennensis* (Rich.) Vahl - plants that are part of the Amazonian phyto-pharmacopeias. For the informants, backyards also encompass areas of surrounding undergrowth clearing and oligarchical forests, which are forests dominated by up to three species that produce fruits and seeds of economic importance and extend over a wide area. In these areas, there is continuous management of species such as *B. excelsa* and *Theobroma grandiflorum* (Willd. ex Spreng.) K. Schum. Amazonian trees are found predominantly in the upland forest area, such as *Copaifera epunctata* Amshoff, *Ormosia coutinhoi* Ducke, and *Aspidosperma nitidum* Benth. ex Müll.Arg. Plant parts are extracted from these species periodically, mainly during extractivism, hunting, and fishing activities. The emphasis on the use of species obtained from the terra firme forest areas may be related to the fact that this area cover more than 90% of the local forest potential compared to the floodplain and alluvial dense ombrophilous lowland (Lisboa et al., 2013).

The species that make up the local collection are found spontaneously in natural or anthropically modified environments (50%) or through cultivation (50%) (Supplementary Table 1). The frequency of use of spontaneous plants is expected in areas with high availability of plant material in natural environments and that are seasonally affected by periods of flooding, which hinders the cultivation of different species (Sarquis et al., 2019; Pedrollo et al., 2016; Rocha et al., 2013). In the present study, however, it was noted that the informants are concerned about maintaining a collection of more accessible plants in their backyards, using “jiraus” (a kind of fence made of sticks) that protect herbaceous species from floods and animal attacks, as well as by planting shrubs and trees in dryland spaces near residences that are not affected by floods (Figure 3). *Alternanthera ficoidea* (L.) P. Beauv. and *Bacopa monnierioides* (Cham.) B.L.Rob., *Hyptis crenata* Pohl ex Benth are examples of naturally present species in igapós. However, these are also cultivated because of their importance in local healthcare.

The various plant parts make up the traditional preparations, with leaves (29%), stem bark (20%), and roots (9%) standing out. Although the leaves are the most used organs in this community, the stem bark stands out when it comes to the use of tree species, specially due to the ease of access or for their organoleptic characteristics, visually associated with the therapeutic properties attributed to them. The barks of *A. nitidum*, *Geissospermum argenteum*, and *Homalolepis cedron*, whose bitter taste is attributed to efficacy in treating malaria and high fevers, are cited as examples. Similar results were observed in other Amazon communities (Coelho-Ferreira, 2009; Pedrollo et al., 2016).

Although the use of plant parts that can endanger the existence of specimens, such as the bark, bast, roots, and stems of tree species, has been registered, there is a noticeable local concern about not exhausting these resources, aiming at their continuous use.

With regard to preparation methods, a total of 679 recipes were recorded, 84% of which were prepared using a single plant. However, depending on the type of disease and symptom treated, they can rely on several species, including animal parts (Supplementary Table 1). In general, considering the parts used and applications, it was possible to group the reported recipes into 14 different preparation modes. The most commonly employed were teas obtained by decoction (41%), juices (20%), and plants *in natura* (10%). The medicines are used in several ways, with oral (60%), topical (18%), and baths (16%) being the most frequent. Teas obtained by decoction are prepared for immediate oral consumption. They are bottled and consumed over a week when made with a mixture of barks and roots from different species. Juices are obtained from crushed leaves or rubbed bark and used orally or topically. In addition to juices, topical preparations are made by applying *in natura* vegetable parts such as leaves, oils, resins, and plasters. The baths are indicated according to the body part intended to be treat. They can be a head bath, full body bath, foot bath, and cleansing bath. The use of vegetable juices is also frequent in another community of this National Forest (Rocha et al., 2013), indicating a possible cultural pattern for using certain plants shared by the populations of this Sustainable Use Conservation Unit.

Head baths are prepared by aqueous or alcoholic maceration of herbaceous species, such as *B. monnierioides*, *Conobea scoparioides* (Cham. and Schltdl.) Benth., and *Bignonia nocturna* (Barb.Rodr.) L.G.Lohmann, to treat especially colds, headaches, fevers, respiratory problems, and body exhaustion. The body bath combats mainly itching and high fevers by using, for example, *Pentaclethra macroloba* (Willd.) Kuntze and *Pachira aquatica* Aubl., respectively. The bath prepared with the bark of the trunk of the latter species treats a fever known locally as “tiriça”, recognized as a severe fever with a sore throat.

According to the Use Value index, the plants most relevant are *Dalbergia monetaria* L. f. (0.8), *B. excelsa* (0.75), *Carapa guianensis* Aubl. (0.77), *C. epunctata* (0.71), and *Citrus limon* (L.) Osbeck (0.68) (Table 2). The use of *C. guianensis* and *C. epunctata* oils in traditional Amazonian medicine is millennia old, data supported by studies confirming their anti-inflammatory, antifungal, and antimicrobial activities, as well as the therapeutic potential of their main chemical constituents (Arruda et al., 2019; Dias et al., 2023). Similarly, *B. excelsa,* whose fruits are edible, have nutritional and therapeutic properties widely recognized worldwide. Although *D. monetaria* has been used for generations, it has not yet been subjected to conclusive studies on its therapeutic potential and chemical composition. Most of these plants occur in local forest areas and should be targeted for conservation strategies.

**TABLE 2:** The Fidelity Value (FL) and Use Value (UV) attributed to plants species cited by at least three informants in the Community of São Sebastião de Marinaú, Pará, Brazilian Amazon. Fce: citation frequency; FceUP: main use citation frequency.

Plant species	Fce	Main use	FceUP	FL	UV
*Dalbergia monetaria* L.f	25	woman’s inflammation	15	80.00	0.80
*Carapa guianensis* Aubl	18	scabies	11	57.89	0.77
*Bertholletia excelsa* Bonpl	13	chilblain	10	55.5	0.75
*Copaifera epunctata* Amshoff	15	woman’s inflammation diarrhoea, belly ache	10	66.67	0.71
*Citrus limon* (L.) Osbeck	24	influenza	21	87.05	0.68
*Allium sativum* L	16	influenza	5	31.25	0.55
*Scutellaria agrestis* A.St.-Hil. ex Benth	16	earache	16	100.00	0.55
*Anacardium occidentale* L	17	diarrhoea	8	53.33	0.55
*Aloe vera* (L.) Burm. F	15	erysipela	6	40.00	0.40
*Acmella oleracea* (L.) R.K. Jansen	15	stomachache	12	80.00	0.40
*Allantoma lineata* (Mart. ex O.Berg) Miers	17	influenza	17	100.00	0.37
*Psidium guajava* L	15	diarrhoea, belly ache	10	66.66	0.33
*Mangifera indica* L	13	wounds	7	53.85	0.31
*Mentha pulegium* L	12	flatulence	4	33.33	0.31
*Alternanthera ficoidea* (L.) P.Beauv	12	menstruation excessive	6	50.00	0.31
*Cichorium endivia* L	11	Influenza	5	41.66	0.31
*Hyptis crenata* Pohl ex Benth	13	stomach each	9	81.81	0.31
*Costus* sp	12	kidney sotnes, urinary infection/inflammation	8	80.00	0.31
*Kalanchoe pinnata* (Lam.) Pers	12	erysipelas	6	50.00	0.28
*Himatanthus articulatus* (Vahl) Woodson	9	genital female complain	3	37.50	0.28
*Fridericia chica* (Bonpl.) L.G.Lohmann	10	anemia	10	100.00	0.24
*Petiveria alliacea* L	6	headache	3	50.00	0.24
*Jatropha gossypiifolia* L	6	wounds	3	50.00	0.24
*Ayapana triplinervis* (M.Vahl) R.M.King and H.Rob	10	quebrante (chipping)	5	50.00	0.22
*Montrichardia linifera* (Arruda) Schott	10	pneumonia	7	70.00	0.22
*Zingiber officinale* Roscoe	10	influenza, cough	6	60.00	0.22
*Phyllanthus niruri* L	10	kidney problems	10	100.00	0.22
*Byrsonima crassifolia* (L.) Kunth	10	wounds	5	50.00	0.22
*Citrus × aurantium* f. *aurantium*	9	palpitation	4	44.44	0.22
*Ruta graveolens* L	7	headeach	5	57.14	0.22
*Dianthera calycina* (Nees) B.D.Jacks	8	anemia	8	100.00	0.22
*Jatropha curcas* L	7	influenza	4	50.00	0.22
*Quassia amara* L	7	malaria	7	100.00	0.22
*Rolandra fruticosa* (L.) Kuntze	7	malaria	3	42.85	0.22
*Geissospermum argenteum* Woodson	7	malaria	4	57.14	0.22
*Syzygium cumini* (L.) Skeels	8	diarrhoea	6	75.00	0.17
*Scoparia dulcis* L	8	prickly heat	8	100.00	0.17
*Brassica oleracea* L	8	vomiting	4	50.00	0.15
*Conobea scoparioides* (Cham. and Schltdl.) Benth	6	influenza	4	66.66	0.15
*Bixa orellana* L	6	pneumonia	6	100.00	0.15
*Dysphania ambrosioides* (L.) Mosyakin and Clemants	6	worms	3	50.00	0.15
*Aspidosperma nitidum* Benth. ex Müll.Arg	6	malaria	4	66.66	0.15
*Connarus perrottetii* var*. angustifolius* Radlk	5	woman’s inflammation	4	60.00	0.15
*Pentaclethra macroloba* (Willd.) Kuntze	6	snakebites	3	50.00	0.15
*Virola surinamensis* (Rol. ex Rottb.) Warb	5	diarrhoea	4	66.66	0.13
*Bacopa monnierioides* (Cham.) B.L.Rob	12	Influenza	6	50.00	0.31
*Euterpe oleracea* Mart	8	diarrhoea	3	37.50	0.24
*Annona exsucca* DC.	9	malaria	9	100.00	0.22
*Gymnanthemum amygdalinum* (Delile) Sch.Bip. ex Walp	8	stomachache	5	62.05	0.22
*Ananas comosus* (L.) Merr	5	worms	3	60.00	0.15
*Musa paradisiaca* L	5	toothache	3	50.00	0.13
*Gossypium arboreum* L	5	chilblain	2	40.00	0.13
*Spondias mombin* L	5	stingray wounds and wounds	3	60.00	0.13
*Parahancornia fasciculata* (Poir.) Benoist	5	bronchitis	3	60.00	0.11
*Theobroma grandiflorum* (Willd. ex Spreng.) K.Schum	5	diarrhoea	3	60.00	0.11
*Uncaria guianensis* (Aubl.) J.F.Gmel	5	woman’s inflammation	3	60.00	0.11
*Eleutherine bulbosa* (Mill.) Urb	5	diarrhoea	3	60.00	0.11
*Manilkara elata* (Allemão ex Miq.) Monach	5	diarrhoea	4	80.00	0.11
*Anacardium giganteum* W.Hancock ex Engl	5	woman’s inflammation	5	100.00	0.11
*Passiflora edulis* Sims	5	feeling anxious	5	100.00	0.11

### 3.3 Faunal resources

**FIGURE 4:**
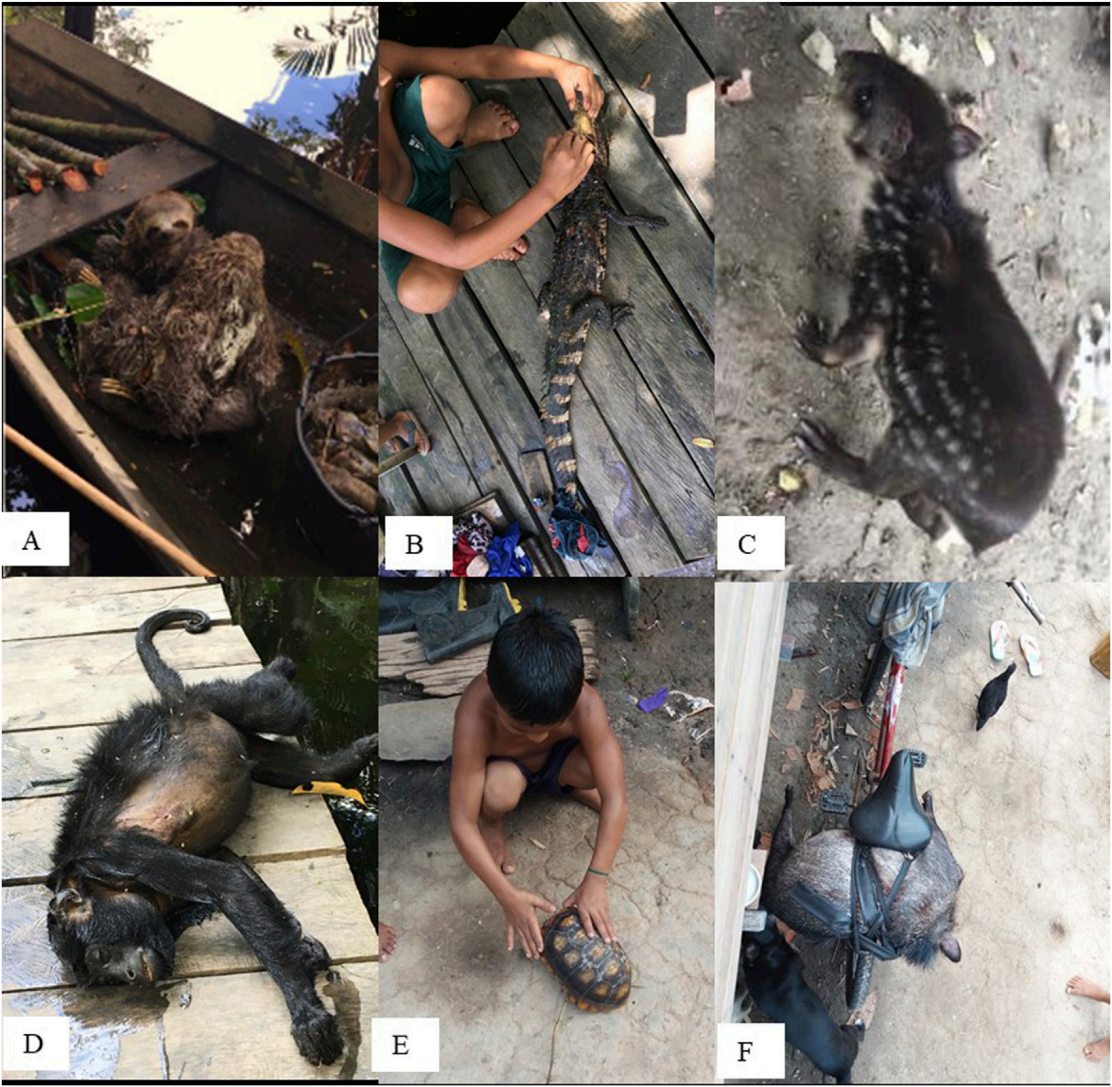
Some of the wild animals consumed by residents of the São Sebastião de Marináú Community, Flona de Caxiuanã, Pará, Brazilian Amazon. **(A)** sloths, **(B)** caiman, **(C)** paca, **(D)** monkey, **(E)** tortoise, **(F)** wild pig.

Residents of Marinaú reported 58 citations associated with at least 21 animals that are found in the CNF. All mentions refer to vertebrates, classified as fish (8), reptiles (5), birds (3), mammals (3), and amphibians (1) (Table 3). These animals are among those most consumed in the local diet, either through hunting, fishing, or domestic breeding, as is the case with chickens and pacas (Figure 4). The use of vertebrates in local medicine by traditional communities has been reported in other Sustainable Use Conservation Unit in Brazil. This can be justified by the reality of populations living in relative isolation and consequent daily contact with animal resources, which enables the utilitarian recognition of these organisms, including those that pose some risk, such as venomous animals (Torres et al., 2009).

**TABLE 3:** Animals from which parts are removed to be used in therapeutic preparations in the Community of Marinaú, Pará, Brazil. Legend: UR: use-reports.

Scientific name	Venacular name	Indicação	Parts used	Application	UR
*Bradypus variegatus* (Schinz, 1825)	preguicinha benta	stroke	fat	topical	3
*Cairina moschata* (Linnaeus, 1758)	pato-preto	garrotilho	fat	oral	2
*Crocodilurus amazonicus* SPIX, 1825	jacarena	“piema”	fat	oral	1
*Cuniculus paca* Linnaeus, 1766	paca	earache, inflammation, snakebites, pneumonia, stingray wounds	fat, gall	oral, topical	8
*Curimata* sp	curimatã	malaria	gall	oral	1
*Cyphocharax* sp	curimatã	malaria	gall	oral	1
*Dracaena guianensis* DAUDIN, 1801	calango, jacuraru	remove thorns from the body	fat	topical	1
*Electrophorus electricus* (Linnaeus, 1766)	poraquê	lice	spine	topical	2
*Gallus domesticus* (Linnaeus, 1758)	galinha	snakebites, stingray wounds, swellings, erysipela, boil, earache, garrotilho	eggs, fat, blood	topical, oral	8
*Hoplias malabaricus* (Bloch, 1794)	sulambra/traíra	earache	fat, gall	topical	7
*Myrmecophaga tridactyla* Linnaeus, 1758	tamanduá-bandeira	snakebites, “susto” (fright)	fat, fur	topical, smoking	3
*Paratrygon aiereba* (Müller and Henle, 1841)	arraia-aramaça	swellings	fat	topical	1
*Podocnemis* sp	tartaruga	stingray wounds	fat	topical	1
*Polychrus marmoratus* (Linnaeus, 1758)	iguana/camaleão	stingray wounds, snakebites	fat	topical	2
*Potamotrygon constellate* (Vaillant, 1880)	arraia-tinga	swellings	fat	topical	1
*Potamotrygon motoro* (Müller and Henle, 1841)	arraia-preta	swellings	fat	topical	1
*Sapajus apela* (Linnaeus, 1758)	macaco-prego	male impotence, chickenpox, pulled muscle, fracture	penis, fat, bones	oral, cataplasm	7
*Sotalia fluviatilis* Gervais, 1853	boto	stroke, garrotilho	fat	topical	3
*Melanosuchus niger* (Spix, 1825)	jacaré-açú	wounds, snakebites, “derrame”, stingray wounds, “piema”, remove thorns from the body	fat, penis	topical, oral	5
Not determined	sapo-arú	“susto” (fright)	resin	smoking	2

The mentioned fish diversity highlights its importance for the subsistence of these populations since several cultural, social, and economic aspects of riverine communities are characterized by the wide availability of these resources in the rivers of this region. Thus, conserving these animals is a matter of concern for the residents of this community, who seek not to exhaust this animal resource. For this purpose, they have established internal rules on the use of fishing tools, such as ‘zagaias’ — wooden arrows thrown to capture fish — instead of fishing nets.

The most important animals were paca - *Cuniculus paca* Linnaeus, 1766, capuchin monkey - *Sapajus apella* (Linnaeus, 1758), traíra - *Hoplias malabaricus* (Bloch, 1794), chicken - *Gallus domesticus* (Linnaeus, 1758), and alligator - *Melanosuchus niger* (Spix, 1825) (Table 4). Since these animals are consumed as food, the parts not used this way are more commonly applied in medicinal recipes. This is the case with the paca, from which the gall and lard are removed during preparation. Later, they are used to treat pneumonia and snakebites. This animal was also the animal wich presented the highest use value among the others (Table 4). The paca was also the most cited animal in the study investigating animals employed in therapeutic practices in Bahia, where reported uses include snakebites (Teixeira et al., 2020).

**TABLE 4:** The Fidelity Value (FL) and Use Value (UV) attributed to natural animal spcies cited by at least, 10% of adults informants in the Community of São Sebastião de Marinaú, Pará, Brazilian Amazon. Fce: citation frequency; FceUP: main use citation frequency.

Nome científico	Fce	Main use	FceUP	FL	UV
*Cuniculus paca* Linnaeus, 1766	8	pneumonia	4	50	0.31
*Hoplias malabaricus* (Bloch, 1794)	7	earache	7	100	0.21
*Sapajus apela* (Linnaeus, 1758)	7	pulled muscle, fracture	5	71,42857	0.25
*Gallus domesticus* (Linnaeus, 1758)	8	garrotilho	2	25	0.25
*Melanosuchus niger* (Spix, 1825)	5	stroke	2	40	0.18
*Bradypus variegatus* (Schinz, 1825)	3	stroke	3	100	0.09
*Myrmecophaga tridactyla* Linnaeus, 1758	3	snakebites	2	66.66667	0.09
*Sotalia fluviatilis* Gervais, 1853	3	stroke	2	66.66667	0.09

Animal parts reported in the Marinaú community are used to treat different symptoms and diseases distributed in six categories of the ICPC: digestive, dermal, respiratory, musculoskeletal systems, culture-bound syndromes, infectious diseases, and ear diseases (Table 5). The most commonly treated problems are snakebites, strokes, fractures, and stingray stings. These conditions and the animals reported here appear in other research on the therapeutic practices of Amazonian communities (Santos et al., 2012; Silva, 2008).

**TABLE 5:** Diseases and symptoms reported for categories of the International Classification of Primary Care (ICPC) in addition to cultural syndromes and the Informant consensus factor (ICF) of medicinal use, as indicated by riverine in the São Sebastião de Marináú Community, Pará, Brazil.

Disease categories (ICPC)	Number of taxa (plants*, animals **)	Number of citations	ICF	Therapeutic indications (number of citations)
Digestive	44*	213	0.80	amoeba D96 (5), worms D96 (16), gastritis D73 (9), belly ache D01 (16), stomach each D02 (50), diarrhoea D11 (84), vomiting D10 (8), liver problems D97 (4), toothache D19 (9), stomach injury D80 (1), indigestion D07 (2), hepatitis D72 (1), vesicule stones D99 (1), sapinho (3), flatulence D08 (7), hiccough (1)
Skin	42*, 13 **	187	0.71	laceration nail S18 (1), wounds S19 (45), stingray wounds S13 (12), garrotilho R83 (5), snakebites S19 (20) scabies (28), erysipela S11 (20), chilblain S74 (12), furuncle S99 (5), Impingem S74 (8), prickly heat (11), pruritus S02 (3), “coceira de imbiara” (1), lice S12 (5), skin yeast infections S74 (2), dandruff (1), burn S14 (3), swellings S05 (5), remove thorns from the body (1)
Respiratory	59*, 2**	148	0.59	influenza R80 (78), pneumonia R88 (30), sore throat R21 (5), cough R05 (10), phlegm abnormal R25 (11) hoarse throatt/voice R29 (3), bronchitis R78 (3), “piema (asthma R96) (6), tired feeling (1), nasal congestion (2)
Female genital	23*	84	0.73	Menstruation excessive X06 (11), menstrual pain X02 (11), woman’s inflammation (25), uterine cancer X77 (2), ovary cysts X77 (1), menstruation absent X05 (3), ovarian cancer X81 (1), mioma (3), menstruation irregular X07 (1), Inflammation in the uterus X29 (1)
Cultural syndromes	17*, 5**	55	0.61	quebrante (chipping), (8), strong fever “tiriça” (3), “susto” (fright) (5), clean the body (1), “wind in baby’s navel” (flatulence) (4), “wind in the gut” (1), “mau olhado” (evil eye) (1), “panemice” (contaminated, unhealthy, unlucky) (4), feeling anxious (7), stubborness (3), “unruly child” (1), “derrame” stroke (12)
Infectious diseases	16*, 2**	51	0.63	measles (4), leishmaniasis (8), malaria (32), oral thrush (3), chickenpox (1), tuberculosis (2), dengue (2)
Musculoesquelético	17*, 1**	42	0.59	fracture L73 (4), “quebradura” (fracture L76) (2), rheumatism L88 (10), joint pain L20 (6), pulled muscle (2), “open chest” (1), bone pain L29 (7), back pain L02 (8), leg pain L14 (1)
Ear	10*, 3**	41	0.70	earache H01 (37)
Urological	11*	31	0.67	urinary calculus U95 (7), urinary infection U29 (15), painful urination U01 (4), kidney problems U14 (5)
Neurological	15*	28	0.48	headeach N01 (27), dizziness N17 (1)
General and Unspecified	16*	24	0.35	bleeding A10 (3), weakness A04 (6), chills A02 (2), fever A03 (5), pain body A01 (2), inflammation A29 (6)
Blood, Blood Forming Organs and Immune Mechanism	3*	18	0.88	Anemia B82 (19)
Male Genital	10*	14	0.31	prostate complains Y06 (10), male impotence Y07 (5)
Cardiovascular	7*	12	0.45	hypertension k25 (4), heart complains X29 (3), palpitation K04 (5)
Pregnancy Childbearing, Family Planning	4*	5	0.25	*postpartum* bleeding W17 (1), *postpartum* cleaning W70 (3), unblocking milk ducts (1)
Eye	1*	1	0.00	eye complaint F29 (1)
Endocrine/Metabolic and Nutritional	1*	1	0.00	diabetes T90 (1)

Lard was mentioned in most of the recipes using animal resources. In general, the animals are boiled or roasted for consumption, and during this process, the exceeding lard is separated, stored, and used as medicine for months. Lard, in general, is applied topically or as a plaster when mixed with vegetable and/or animal parts. Some animal parts are used in the composition of teas and bottled products together with plant parts, to treat, for example, erectile dysfunction. In this case, the genitals of monkeys and alligators are used along with the roots of *Ptychopetalum olacoides* Benth. Another example is alligator lard, which, when mixed with the leaves of the *Cissus verticillata* (L.) Nicolson and C.E. Jarvis, forms an ointment that is applied to rub the body parts affected by the stroke.

The use of animals either for food or in local medicine in traditional communities brings to light several issues involving the conservation of these organisms. Among those mentioned, it was observed that two species are on the Official List of Species of Brazilian Fauna Threatened with Extinction: *Myrmecophaga tridactyla*, which is considered vulnerable, and *Paratrygon aiereba*, critically endangered (Ministério do Meio Ambiente, 2022). This information reinforces the area’s importance from a biological point of view and highlights the need to establish conservation strategies, such as encouraging the breeding of animals involved in therapeutic and food practices.

### 3.4 Diseases and symptoms treated and consensus of use

The classification of diseases and symptoms in communities with a traditional system as a priority, and in many cases unique in healthcare, is somewhat complex. In this study, it was possible to observe that the terminologies used for therapeutic indications, in most cases, resemble the medical-scientific vocabulary, which may have been influenced by contact with professionals in the Ferreira Penna Scientific Station ward or sporadically with Western medical services in the city. Other times, they are diseases whose causes, diagnosis, and treatment are perceived according to local cultural concepts.

One hundred sixteen mentioned diseases and symptoms were classified in 18 of the 19 ICPC categories (Table 5). Concerning the number of mentions, those of the digestive, respiratory, and dermal systems stood out. These categories comprise 44% of the total symptoms and diseases treated in this community and are frequently recorded at the top of the list of health problems reported in ethnobotanical research in the Brazilian Amazon (Bieski et al., 2015; Sarquis et al., 2019; Pedrollo et al., 2016).

Diarrhea was this community’s most recurrent health problem, accounting for 60% of the digestive system citations. This gastric disorder was also highlighted by Rocha et al. as well in another community in this National Forest. Furthermore, it appeared as the main cause of hospitalizations according to data from the latest IBGE census in the municipality of Portel (Governo Federal do Brasil, n. d.), where the community under study is located. Based on the observations made during the visits, the frequency of diarrhea is believed to be related to the conditions in which people live, that is, without access to safe drinking water and adequate sewage systems.

With regard to the Informant Consensus Factor (ICF) about the use of local resources, 11 of the 18 categories presented values equal to or greater than 0.50. The most important were diseases of the blood (0.88), digestive system (0.80), female genital system (0.73), dermal system (0.70), and ear (0.7) (Table 5). The main aspects of these five categories will be discussed below, as well as the species with the highest Fidelity Level (FL%) values used in each category (Tables 2, 4).

The blood disease category seems to comprise only anemia in Marinaú. An anemic person is pale, weak, and oversleeps. Of the three species used in this category, *F. chica* (Bonpl.) L.G. Lohmann and *Dianthera calycina* (Nees) B.D.Jacks are the species used by all informants (FL = 100%). Pariri is a native Amazonian liana with a wide distribution in Brazil. The use of this species for anemia goes back generations in the Amazon region (Matias et al., 2021). Anemia has two main causes: nutritional, especially due to micronutrient deficiency, such as Fe, and infectious, as in the case of malaria. Dos Santos Magalhães et al. (Magalhães et al., 2009) observed that Fe was the most abundant element in the dried leaves of *Fridericia chica*. In addition, the presence of flavonoids, saponins, alkaloids, and phenolic compounds with biological activities in this species has been observed, as well as its antitumor, anti-inflammatory, antimicrobial, photoprotective, and regenerative potential (Matias et al., 2021).

Diseases of the digestive system are treated with 44 species; the main problems are diarrhea, abdominal pain, and stomach ache. *Allantoma lineata* (Mart. ex O. Berg) Miers was the plant with the most reported uses, especially to treat diarrhea (FL = 100%). According to the informants, the juice of this species helps to contain water loss in the feces, reducing dehydration, besides killing possible worms that cause it. The ceruzeiro is a large tree of the Lecytidaceae family, endemic to the Amazon and found along riverbanks. Rocha et al. (2013) also reported using the species to treat this disease in the National Forest. No other reports of use in the Amazon were found in the literature consulted, nor were any other chemical and biological studies of therapeutic evaluation. The high consensus on this plant’s use is relevant to further studies. In addition, several species of the aforementioned family traditionally used for the same therapeutic purpose have been researched; the results have supported their traditional uses (Ferreira et al., 2021).

The consensus observed on using plants for treating diseases of the female genital system may be associated with the more significant number of women participating in this research. However, it was noted that many of the interviewees provided information with a certain amount of embarrassment. Thus, the generalist term “inflammation of women” was the most frequently mentioned, followed by menstrual cramps and irregular menstruation*. D. monetaria* received the highest consensus of use in this category, especially for treating inflammation of women (FL = 80%). It is a native liana, often found in the floodplain Amazon estuary (Ferreira et al., 2005). Amazonian populations use it mainly to treat female genitourinary system problems, anemia, and gastric diseases (Melo et al., 2022). Its therapeutic potential is locally associated with the reddish coloration of its bark and astringent flavor. Phyto-chemical studies have verified the anti-inflammatory and antibacterial activity of extracts obtained from the bark of this species, besides the presence of saponins, phenols, and proanthocyanidin-type tannins. They also act as antibacterial, antidiarrheal, hemostatic, and healing agents (de Moura et al., 2020).

Wounds from injuries or accidents caused during the informants’ daily activities were the most frequently mentioned problems in the dermal system category, in addition to other diseases caused by insect and snakebites and stingray stings. Most of the animals cited in Table 3 are for treating diseases of this system, being snakebites the most commonly treated symptom with the lard of *C. paca*, *M. tridactyla*, *M. niger*, *Polychrus marmoratus,* and *Dracaena guianensis*. During fieldwork, it was observed that snakes are very feared, given their potential lethality and the frequency with which they are found in the study area. The name of this National Forest is justified by this. Regarding the use of plants in this category, the *Scoparia dulcis* L. was the one that obtained the highest consensus to combat heat rash or skin allergy (FL = 100%) and met what was reported by Coelho-Ferreira (2009) and Pedrollo et al. (2016). The rashes can be caused by the hot and humid climate of the region, which mainly affects children. *S*. *dulcis* has been the target of several studies attesting to its anti-inflammatory, antifungal, antiallergic, and antimicrobial activity (Jiang et al., 2021). Its main chemical compounds are scopadulcic acids A and B, scopadiol, scopadulciol, scopadulin, scoparic acids A- C, and betulinic acid (Paul, 2017). The hydroethanolic extract of *S. dulcis* showed antiallergic activity in rats after exposure to a known allergen. The authors attributed this to the presence of tannins, steroids, saponins, alkaloids, and glycosides that act as inhibitors of histamine, bradykinin, and serotonin release from inflammatory cells (Ofori-Amoah et al., 2016).

Earache was the only symptom in the last category highlighted, for which a collection of ten plant species and three animal species were indicated. Informants report that the cause of this symptom may be related to bathing in the river, which is part of the daily routine of riverine communities. This activity favors the proliferation of microorganisms that cause otitis, an ear infection or inflammation. *Scutellaria agrestis* A.St.-Hil. ex Benth., which showed the highest consensus in this category (FL = 100%), is a native herb widely cultivated in backyards of this and other Amazonian riverine communities (Bieski et al., 2015; Sarquis et al., 2019). The preliminary study by Oliveira et al. (2014), conducted with Wistar rats, showed that the aqueous extract of *S. agrestis* leaves showed anti-inflammatory, analgesic activity, and little toxicity. As for animal resources, the lard or gall of *H. malabaricus* also presented FL = 100% for treating earache (Table 4). The use of this resource for the same purpose was also recorded in other communities in Brazil as well (El-Deir et al., 2012; Silva, 2008).

In addition to these categories, cultural-bound syndromes are worth mentioning, given their local importance. “Derrame” (stroke), “quebrante” (chipping), “susto em criança” (child fright), “panemice” (contaminated, unhealthy, unlucky), and “mau olhado” (evil eye) are the most cited problems, treated with baths and smokings made with a mixture of plant and animal parts. During the preparation of medicines for these purposes, elements such as the moon’s phases and the sun’s heat are considered, and prayers, rituals, and chants accompany the applications. They are usually recognized by the elderly and can be confused with other diseases. Some of the clinical manifestations of these diseases can also be recognized by conventional medicine (Pagani et al., 2017), for example, “derrame”, which in Marinaú, is a disease caused by a bad wind or the visit of an evil spirit, and is recognized by the loss of facial and motor movements, fever and tremors, symptoms that can be clinically associated with a cerebral cardiovascular accident. It indicates that natural resources used to treat these diseases should not be neglected in further studies, mainly because species such as *Mansoa alliacea* (Lam.) A.H.Gentry has been historically recorded among Amazonian populations to treat strokes (Coelho-Ferreira, 2009; Pagani et al., 2017; Santos et al., 2012).

## 4 Conclusion

The São Sebastião de Marinaú community is included among the diverse riverine populations that live in relative isolation in the Amazonian forests and hold a diverse, rich, and highly consensual knowledge of floristic and faunal uses in daily healthcare. The ethnobiological knowledge obtained is well distributed among the different age groups and genders and is relatively high when compared to the wealth of medicinal resources recorded in other Amazonian riverside communities. This can be attributed to the daily activities shared among individuals of varying genders and ages within the community. The plants and animals mentioned show the importance of native natural resources for the subsistence of this community. The richness of this knowledge can be attributed to its cultural heritage, which reflects the influences of African-American, indigenous, and white ancestries that shaped the community’s identity. This, combined with geographical isolation, unmet health needs that official medicine could not address, and proximity to the abundant resources of the Amazon rainforest, has contributed to the development of a unique health disease system.

In the community of Marinaú, there are no adequate sewage systems, and the water consumed generally comes from rivers. This facilitates the development of gastrointestinal diseases. In addition, there is no healthcare in the local community. The combination of these two factors makes plants a priority resource for digestive healthcare. The species with the most citations in the category of digestive diseases are predominantly native, of wide local availability, and traditionally used in the Amazon. Among the species with the highest consensus of use among informants, the *A. lineata* stands out, furthermore, there are only reports of medicinal use of this species in the CNF. The local agreement on using this plant’s bark to treat diarrhea encourages further ethnobotanical, phyto-chemical, and biological research on it. Snakebites are a severe health problem and are treated with animal parts, especially the gall and lard of the *C. paca*. Animal therapy is a common practice among the residents and highlights the need for further studies in the CNF, not only to document this knowledge but also to develop conservation strategies.

This study underscores the vital role of traditional communities in sustainable conservation units, as their involvement is crucial for preserving plant and animal species essential to local traditional medicine. Additionally, mapping the occurrence and use of species in vulnerable conditions aids in developing effective conservation strategies for these resources. Such research also promotes the recognition of non-timber products as valuable raw materials with potential applications in chemical and pharmaceutical industries. Moreover, it serves as a key resource for preserving cultural heritage and enhancing local community engagement in managing and making decisions about the sustainable use of resources in the Caxiuanã National Forest.

## Data Availability

The original contributions presented in the study are included in the article/Supplementary Material, further inquiries can be directed to the corresponding author.
