# Supplementary material for: Natural resources used in the traditional medicine of the Marinaú community, Caxiuanã forest, Brazil
Source: Front Pharmacol. 2024 Oct 9;15:1443360. doi: 10.3389/fphar.2024.1443360 (PMC11496069; doi:10.3389/fphar.2024.1443360)
Supplement: Supplementary file 1 [file Table1.pdf]

Supplementary Table 1. Medicinal plants used by residents of the São Sebastião de Marináú Community, Flona de Caxiuanã, Pará, Brazilian Amazon.

Family/species	Voucher	Vernacular names	Growth form	Traditional uses (citation number)	Plant part used	Origin	Source	Preparation method	Apllication route
**Acanthaceae**									
Dianthera calycina (Nees) B.D.Jacks.	MG239794	Fosangue	herb	anemia (8), bleeding (1)	leaves	native	gathered	decoction	oral
**Adoxaceae**									
Sambucus nigra L.	MG_Etn_00669	sabugueiro	herb	measles (3)	leaves	exotic	cultivated	decoction	oral
**Amaranthaceae**									
Alternanthera ficoidea (L.) P.Beauv.	MG241229	cuia-mansa, piriquitinho	herb	worms (1), bleeding (1), menstruation excessive (6), menstrual pain (2), post-partum bleeding (1), headache (2), unruly child (1), quebrante (chipping), stubbornness, “mau-olhado” (evil eye)	whole plant	exotic	cult., gat.	Infusion, decoction	bath, oral
Dysphania ambrosioides (L.) Mosyakin & Clemants	MG_Etn_00669	Mastruz	herb	worms (3), gastroenteritis (1), fracture (2)	whole plant	exotic	cultivated	juice	oral, topical

Family/species	Voucher	Vernacular names	Growth form	Traditional uses (citation number)	Plant part used	Origin	Source	Preparati on method	Apllication route
**Amaryllidaceae**									
Allium cepa L.	MG_Etn_00 671	cebola	herb	influenza (1), laceration nail (1)	root	exotic	cultivated	mixture or syrup	oral
Allium sativum L	MG239777	alho	herb	pneumonia (2), influenza (5), ear pain (1), rheumatism (2), joint pain (2), hypertensin (1), worms (1), abdominal ache (2), stomach each (2), throat pain (1)	bark	exotic	cultivated	decoction, syrup, mixture with a coffe, infusion, maceration in alcohol	oral, topical
**Anacardiaceae**									
Anacardium giganteum W.Hancock ex Engl.	MG_Etn_00 653	cajuí, caju-açú	tree	genital female complain (5)	inner bark	native	gathered	decoction	oral
Anacardium occidentale L.	MG_Etn_00 637	cajuzeiro, cajueiro, caju	tree	leishmaniasis (1), skin injury (4), diarrhoea (8), pneumonia (1), abdominal ache (2), menstruation excessive (1), postpartum cleaning (1)	bark, inner bark	native	cult., gat.	*in natura*, syrup, infusion	oral, topical, bath

Family/species	Voucher	Vernacular names	Growth form	Traditional uses (citation number)	Plant part used	Origin	Source	Preparation method	Apllication route
Mangifera indica L.	MG_Etn_00636	mangueira	tree	diarrhoea (5), wounds (7) stingray wounds (1), laceration (1)	inner bark	exotic	gathered	scrape the inner bark until yellow milk appears, mixture this scraping with water, and let it rest. the water is withdrawn and solid used on the wound; the juice mixture with a coffee or water. or, you can also chew the "envira"	topical, oral
Spondias mombin L.	MG_Etn_00648	taperebá	tree	wounds (3), stingray wounds (2)	inner bark, seed	native	cultivated	juice	topical
Tapirira guianensis Aubl.	MG_Etn_00667	tatapiririca		throat pain (1)	bark	native	gathered	juice	oral

Family/species	Voucher	Vernacular names	Growth form	Traditional uses (citation number)	Plant part used	Origin	Source	Preparation method	Apllication route
**Annonaceae**									
Annona exsucca DC.	MG239777	envira-preta	tree	malaria (9)	inner bark	native	gathered	maceration	bath
Annona mucosa Jacq.	MG241232	briribazeiro	tree	garrotilho (2), wounds (1)	bark	native	cultivated	mixture with a animal fat, decoction	oral
Duguetia riparia Huber	MG_Etn_00 658	envirataia	tree	rheumatism (2)	bark	native	gathered	infusion	bath
Fusaea longifolia (Aubl.) Saff.	MG241240	carniceira	tree	snakebites (2)	bark	native	gathered	maceration	oral
**Apocynaceae**									
Aspidosperma nitidum Benth. ex Müll.Arg.	MG_Etn_00 661	carapanã	tree	malaria (4), repelente (2)	bark	native	gathered	infusion	bath
Couma guianensis Aubl.		sorva	herb	diarrhoea (1)	leaves	native	gathered	decoction	oral
Geissospermum argenteum Woodson	MG239793	taquarirana, pau-pereira	tree	malária (4), curuba (3), repelent	bark	native	gathered	maceration	bark
Himatanthus articulatus (Vahl) Woodson	MG_Etn_00 654	sucuúba	tree	cough (2), influenza (2), pneumonia (2), phlegm abnormal (2), erysipela (1), pulled muscle (1), “open chest” (1), menstrual pain (1), genital female	bark, latex	native	gathered	decoction, *in natura*	oral

Family/species	Voucher	Vernacular names	Growth form	Traditional uses (citation number)	Plant part used	Origin	Source	Preparati on method	Apllication route
				complain (3), uterine cancer (1), ovary cysts (1), diarrhoea (1)					
Parahancornia fasciculata (Poir.) Benoist	MG_Etn_00 642	amapá-amargo	tree	bronchitis (3), tuberculosis (1), diarrhoea (1)	latex	native	gathered	*in natura* or mixture with a coffe	oral
**Araceae**									
Montrichardia linifera (Arruda) Schott	MG_Etn_00 635	aninga	herb	pneumonia (7), stingray wounds (2)	(1,2) leaves; (2) bark	native	gathered	roasted and mixture with a bee honey it is burned and placed on the wound	oral, topical
**Arecaceae**									
Acrocomia aculeata (Jacq.) Lodd. ex R.Keith		mocajá	tree	gastritis (1)	root	native	gathered	decoction	oral
Attalea maripa (Aubl.) Mart.		anajá	tree	earache (3)	seed	native	gathered	sifted and mixture with a water	topical
Cocos nucifera L.		coqueiro	tree	weakness (3)	fruit	native	cult., gat.	*in natura*	oral
Euterpe oleracea Mart.		açaizeiro	tree	chills (2), diarrhoea (3), vomiting (1), worms (1), cut	root, bark, fruit, leaves	native	cult., gat.	decoction; roast the heart of the palm,	oral, topical; intravaginal; bath

Family/species	Voucher	Vernacular names	Growth form	Traditional uses (citation number)	Plant part used	Origin	Source	Preparation method	Apllication route
				(1), menstruation absent (1), cancer in the uterus (1)				squeeze, and use the resulting water; powdered the fruit with water and applie in the woman's genitalia for 5 minutes with her legs up; maceration	
Mauritia flexuosa L.f.		miriti	tree	chilblain (3), snakebites (1)	resin	native	gathered	*in natura*	topical
**Asteraceae**									
Acmella oleracea (L.) R.K. Jansen	MG239778	jambú	herb	stomachache (12), liver problems (1), gastritis (2), toothache (1), prostate problems (1), urinary infection (1)	leaves, flowers	native	cult., gat.	decoction	oral
Ayapana triplinervis (M.Vahl) R.M.King & H.Rob.	MG_Etn_00672	japana	herb	influenza (1), diarrhoea (3), quebrante	leaves	native	cultivated	decoction	oral

Family/species	Voucher	Vernacular names	Growth form	Traditional uses (citation number)	Plant part used	Origin	Source	Preparation method	Apllication route
				(chipping) (5), “susto” (fright) (1)					
Centratherum punctatum Cass.	MG241235	melhoral	herb	headache (3)	fo	native	cultivated	decoction	oral
Cichorium endivia L	MG_Etn_00 673	xicória	herb	influenza (5), headache (1), bone pain (1), stomachache (2), worms (1), fever (1)	leaves, root	exotic	cultivated	decoction, maceration of the mixture with alfavaca, peixinho, vick and pataqueira (for 24 hours) maceration in alcohol	oral, bath, topical
Elephantopus mollis Kunth	MG241244	língua-de-vaca	herb	phlegm abnormal (2), influenza (1), hoarse throat/voice (1)	leaves	native	gathered	syrup	oral
Gymnanthemum amygdalinum (Delile) Sch.Bip.	MG_Etn_00 674	Boldo	tree	stomachache (5), stomach injury (1), gastritis (2)	leaves	exotic	cultivated	decoction	oral
Rolandra fruticosa (L.) Kuntze	MG239800	carica-á	shrubby	malaria (3), indigestion (1), vomiting (1), stomachache (1),	root	native	cultivated	decoction	oral, bath

Family/species	Voucher	Vernacular names	Growth form	Traditional uses (citation number)	Plant part used	Origin	Source	Preparation method	Apllication route
				earache (1), pain body (1), menstruation absent (1)					
**Bignoniaceae**									
Bignonia nocturna (Barb.Rodr.) L.G.Lohmann		cipó-curimbó	climber	headache (1)	bark	netive	gathered	maceration in alcohol	bath
Fridericia chica (Bonpl.) L.G.Lohmann	MG_Etn_00 675	Pariri	climber	anemia (10), hepatitis (1), liver problems (1), pneumonia (1)	leaves	native	cult., gat.	decoction	oral
Handroanthus serratifolius (Vahl) S.O.Grose	MG_Etn_00 638	pau-d'arco	tree	menstrual pain (1), woman's inflammation (2), diarrhoea (1)	bark	native	gathered	decoction (see verônica)	oral
Mansoa alliacea (Lam.) A.H.Gentry		cipó-d'alho	climber	"wind in the belly" (flatulence) (2), rheumatism (2), joint pains (1)	leaves	native	cult., gat.	maceration in alcohol, decoction	Topical, oral
**Bixaceae**									
Bixa orellana L	MG239804	urucum	tree	pneumonia (6), asthma (1)	bark	native	cult., gat.	syrup	oral
**Brassicaceae**									

Family/species	Voucher	Vernacular names	Growth form	Traditional uses (citation number)	Plant part used	Origin	Source	Preparation method	Apllication route
Brassica oleracea L.		Couve	herb	vomiting (4), stomachache (1), gastritis (1), wound (1)	leaves	exotic	cultivated	juice mixed with lemon and breast milk, decoction	oral, topical
**Bromeliaceae**									
Ananas comosus (L.) Merr.	MG_Etn_00 676	abacaxi	herb	worms (3), prostate complains (1), vesicule stones (1), urinary infection (1), kidney sotnes (1)	fruit	native	cultivated	decoction, juice	oral
**Caricaceae**									
Carica papaya L.		mamão	tree	worms (3), gastritis (1)	root	exotic	cultivated	decoction alone or mixture with chicória	oral
**Caryocaceae**									
Caryocar villosum (Aubl.) Pers.	MG_Etn_00 639	pequiá	tree	breathing problem (1)	seed	native	gathered	roasting, mash, mixed with water and sifted	oral
**Clusiaceae**									

Family/species	Voucher	Vernacular names	Growth form	Traditional uses (citation number)	Plant part used	Origin	Source	Preparation method	Apllication route
Clusia columnaris Engl.	MG_Etn_00 629	cebola-de-boto	tree	asthma (1), pneumonia (1)	root	native	gathered	syrup	oral
**Chrysobalanaceae**									
Hymenopus macrophyllus (Benth.) Sothers & Prance	MG_Etn_00 666	anoerá	tree	chickenpox (2), inflammation (1), diarrhoea (1), skin allergy (1)	fruit, bark	native	gathered	maceration	topical; oral
**Connaraceae**									
Connarus perrottetii var. *angustifolius* Radlk	MG_Etn_00 677	barbatimão	tree	ovarian cancer (1), menstruation excessive (2), woman's inflammation (4), stomachache (3)	bark	native	gathered	maceration	oral
**Costaceae**									
Costus sp.	MG_Etn_00 678	canafixe	shrubby	kidney sotnes (4), prostate problems (2), urinary infection/inflammation (4), menstruation absent (1), diarrhoea (1)	leaves, bark	exotic	cultivated	decoction	oral
**Crassulaceae**									

Family/species	Voucher	Vernacular names	Growth form	Traditional uses (citation number)	Plant part used	Origin	Source	Preparation method	Apllication route
Kalanchoe pinnata (Lam.) Pers.	MG_Etn_00679	pirarucu-caá, pirarucu	herb	erysipela (6), earache (2), wounds (2), stingray wounds (1), pneumonia (1)	leaves	exotic	cultivated	plaster/cataplasm, juice, syrup	topical, oral
**Cucurbitaceae**									
Cucurbita moschata Duchesne		jerimum	herb	earache (1)	flowers	exotic	cultivated	juice	topical
**Dilleniaceae**									
Davilla nitida (Vahl) Kubitzki	MG241241	cipó-de-fogo, cipó-doce	climber	weakness (3)	bark	native	gathered	*in natura*	oral
**Ebenaceae**									
Diospyros guianensis (Aubl.) Gürke	MG239801	maria-preta	tree	boil (3), impingem (1)	fruit	native	gathered	ointment	topical
**Euphorbiaceae**									
Euphorbia cotinifolia L.	MG241225	leiteiro-vermelho	herb	“panemice” (4)	whole plant	exotic	cultivated	infusion	bath
Euphorbia hirta L.	MG241233	sete-sangria	herb	prickly heat (2)	whole plant	native	cultivated	infusion	bath
Euphorbia tithymaloides L.	MG239779	coramina	tree	heart complains (3)	leaves	native	cultivated	decoction	oral
Jatropha curcas L.	MG239781	pião-branco	tree	influenza (4), toothache (1), wounds (1),	leaves, latex	exotic	cultivated	decoction, *in natura* (latex)	oral, topical

Family/species	Voucher	Vernacular names	Growth form	Traditional uses (citation number)	Plant part used	Origin	Source	Preparation method	Apllication route
				diarrhoea (2), phlegm abnormal (1) oral thrush (2)					
Jatropha gossypiifolia L	MG239782	pião roxo	shrubby	wounds (3), Influenza (1), toothache (2), phlegm abnormal (1), fracture (1), earache (2)	leaves, latex	native	cultivated	infusion, *in naura*, juice with a fat monkey	bath, topical
Manihot esculenta Crantz	MG_Etn_00 680	mandioca	shrubby	pruritus (3), "susto" (fright) (1)	root	native	cultivated	smoking, maceration	smell the smoke, bath, topical
**Fabaceae**									
Anadenanthera peregrina (L.) Speg.	MG_Etn_00 681	angico	tree	influenza (1), phlegm abnormal (1), pneumonia (1), cough R05 (1)	bark	native	gathered	syrup	oral
Copaifera epunctata Amshoff	MG_Etn_00 681	copaiba	tree	menstrual pain (1), woman's inflammation (7), menstruation excessive (2), postpartum cleaning (2), diarrhoea (1),wounds (5),	oil, bark	native	cult., gat.	syrup, *in natura*	oral

Family/species	Voucher	Vernacular names	Growth form	Traditional uses (citation number)	Plant part used	Origin	Source	Preparati on method	Apllication route
				inflammation (1), urinary infection (3), prostate problems (2), influenza (3), pneumonia (4)					
Dalbergia monetaria L.f.	MG_Etn_00 683	verônica	climber	menstrual pain (5), woman's inflammation (15), menstruation excessive (1), postpartum cleaning (2), diarrhoea (6), influenza (2), phlegm abnormal (1), pneumonia (1), cough (1), stomachache (3), myoma (1), clean the body (1)	bark	native	gathered	juice with a coffe, decoction	oral
Diplotropis martiusii Benth.	MG239783	cumarú-do-mato	tree	back pain (3)	seed	native	gathered	maceration in alcohol	oral
Dipteryx odorata (Aubl.) Forsyth f.	MG_Etn_00 684	cumarú	tree	rheumatism (2)	fruit	native	cult., gat.	maceration in alcohol	topical
Hymenaea courbaril L.	MG239333	breu jutaicíca	tree	asthma (2) hoarse throat/voice (1),	resin	native	cult., gat.	smoking	smell the smoke

Family/species	Voucher	Vernacular names	Growth form	Traditional uses (citation number)	Plant part used	Origin	Source	Preparation method	Apllication route
				nasal congestion (2),					
Hymenaea intermedia Ducke	MG_Etn_00 647	jatobá, jutaí	tree	woman's inflammation (4), myoma (1), urinary infection (3), prostate problems (2), worms (1), back pain (3)	bark	native	gathered	maceration	oral
Inga ulei Harms.	MG_Etn_00 627	ingazeiro	tree	wounds (2), oral thrush (1)	bark	native	cult., gat.	juice	topical
Libidibia ferrea (Mart. ex Tul.) L.P.Queiroz	MG_Etn_00 685	Jucá	tree	influenza (1), phlegm abnormal (1), pneumonia (2), cough (1)	bark, seed	native	cultivated	syrup (see babosa), maceration (see lágrimas de nossa senhora)	oral
Machaerium floribundum Benth.	MG_Etn_00 628	juquiri	tree	toothache (3), wounds (2)	latex	native	gathered	*in natura*	oral
Ormosia coutinhoi Ducke		buiuçú	tree	rheumatism (2), bone pain, joint pain (3)	bark	native	gathered	maceration in alcohol	oral, topical
Pentaclethra macroloba (Willd.) Kuntze	MG_Etn_00 649	pracaxi	tree	snakebites (3), wounds (2), sore throat (1),	fruit, oil seed, leaves	native	gathered	the fruits are burned, the ashes	bath, topical

Family/species	Voucher	Vernacular names	Growth form	Traditional uses (citation number)	Plant part used	Origin	Source	Preparation method	Apllication route
				"coceira de imbiara" (1)				are removed, mixed with water, and bathed in the body, *in natura*, juice	
Schnella splendens (Kunth) Vaz	MG239798	escada-de-jaboti	climber	worms (2), diarrhoea (1)	bark	native	gathered	decoction	oral
Swartzia polyphylla DC.	MG239799	pitaica	Tree	toothache (1)	latex fruit	native	gathered	*in natura*	oral
Tachigali paniculata Aubl.	MG_Etn_00 640	taxi-vermelho	tree	leishmaniasis (1)	inner bark	native	gathered	juice	topical
Vatairea guianensis Aubl.	MG239787	faveira	tree	scabies (3), impingem (2)	inner bark, seed	native	gathered	juice	topical
Vouacapoua americana Aubl.	MG_Etn_00 686	Acapu	tree	urinary infection (2), prostate problems (1), back pain (1)	inner bark	native	gathered	decoction	oral
**Hypericaceae**									
Vismia latifolia (Aubl.) Choisy	MG_Etn_00 650	Lacre	ar	impingem (4)	latex	native	gathered	*in natura*	topical
**Humiriaceae**									
Endopleura uchi (Huber) Cuatrec.	MG_Etn_00 645	Uxi	tree	inflammation (1)	bark	native	gathered	decoction	oral
**Iridaceae**									

Family/species	Voucher	Vernacular names	Growth form	Traditional uses (citation number)	Plant part used	Origin	Source	Preparati on method	Apllication route
Eleutherine bulbosa (Mill.) Urb.	MG_Etn_00 687	anajaí, marupaí	herb	influenza (1), worms (2), diarrhoea (2)	leaves, root	exotic	cultivated	decoction	oral
**Lamiaceae**									
Aeollanthus suaveolens Mart. ex Spreng.		caatinga-de-mulata	herb	headache (3)	leaves	exotic	cultivated	infusion	oral
Mentha pulegium L.	MG_Etn_00 688	hortelã	herb	flatulence (4), wind in the gut (1), diarrhoea (1), vomiting (1), belly ache (1), influenza (2), palpitatio (1), feeling anxious (2), quebrante (chipping) (1)	leaves	exotic	cultivated	decoction	oral
Mentha sp.	MG_Etn_00 689	erva-vermelha	herb	menstruation excessive (2)	leaves	-	cultivated	decoction	oral
Mentha sp.	MG239788	Vick	herb	influenza (2)	leaves	-	cultivated	see chicória	bath
Hyptis crenata Pohl ex Benth.	MG239789	salva-do-marajó	herb	stomach each (9), menstrual pain (2), woman's inflammation (1), diarrhoea (1), indigestion (1)	whole plant	native	cult., gat.	decoction	oral

**Family/species**	**Voucher**	**Vernacular names**	**Growth form**	**Traditional uses (citation number)**	**Plant part used**	**Origin**	**Source**	**Preparation method**	**Apllication route**
Ocimum basilicum L.	MG241237	manjericão	herb	stomach each (1), flatulence (1),	leaves	exotic	cultivated	decoction	oral
Ocimum campechianum Mill.	MG_Etn_00689	alfavaca	herb	influenza (2), menstrul pain (1), prostate problems (1), urinary infection (1)	leaves	native	cultivated	decoction	bath, oral
Coleus barbatus (Andrews) Benth. ex G.Don	MG239790	anador	herb	stomach each (2), headache (1), influenza (1)	leaves	exotic	cultivated	decoction	oral
Plectranthus sp.	MG_Etn_00691	boldinho	herb	vomiting (1), stomachache (3)	leaves	exotic	cultivated	decoction	oral
Pogostemon heyneanus Benth.	MG_Etn_00692	Oriza	herb	influenza (1)	leaves	exotic	cultivated	infusion	bath
Pogostemon sp.	MG_Etn_00693	manjerona	herb	cough (1), influenza (1)	leaves	exotic	cultivated	syrup	oral
Scutellaria agrestis A.St.-Hil. ex Benth.	MG239795	trevo-roxo	herb	earache (16)	leaves, latex	native	cultivated	ointment (mixture with jerimumflower and caatinga da mulata leaves)	topical
**Lecythidaceae**									

Family/species	Voucher	Vernacular names	Growth form	Traditional uses (citation number)	Plant part used	Origin	Source	Preparation method	Apllication route
Allantoma lineata (Mart. ex O.Berg) Miers	MG239784	Ceru	tree	diarrhoea (14), headache (1)	bark, inner bark	native	gathered	juice, decoction	oral
Bertholletia excelsa Bonpl.	MG_Etn_00651	castanha-do-pará	tree	chilblain (10), snakebites (3), diarrhoea (5), wounds (2), hypertension (1), anemia (1), malaria (1), menstrual pain (1), belly ache (1), influenza (3)	inner bark, seed, bark	native	gathered	juice, decoction	topical, oral
Eschweilera ovata (Cambess.) Mart. ex Miers	MG241243	matá-matá	tree	stomachache (1)	bark	native	gathered	decoction	oral
**Malpighiaceae**									
Byrsonima crassifolia (L.) Kunth	MG_Etn_00630	muruci	tree	wounds (5), "quebradura" (1), belly ache (1), diarrhoea (1), menstrual pain (1), stingray wounds (1),	inner bark	native	cultivated	juice, infusion	topical, oral
Malpighia emarginata DC	MG_Etn_00695	aceroleira	tree	worms (2)	root	exotic	cultivated	decoction (see piriquitinho)	oral
**Malvaceae**									

Family/species	Voucher	Vernacular names	Growth form	Traditional uses (citation number)	Plant part used	Origin	Source	Preparation method	Apllication route
Hibiscus sabdariffa L.	MG241227	vinagre-de-roxa	herb	influenza (1)	leaves	exotic	cultivated	decoction	bath
Gossypium arboreum L.	MG241238	algodão	tree	chilblain (2), diarrhoea (1), headache (1), menstruation excessive (2), phlegm abnormal (1)	leaves	exotic	cultivated	juice with a honey bee, decoction	oral, topical
Malva sp.	MG_Etn_00694	malva	herb	fear of hypertension (2), eye complaint (1), "tiriça" (severe fever with sore throat) (1)	leaves	exotic	cultivated	syrup	oral
Pachira aquatica Aubl.	MG241230	mamorana	tree	fear of hypertension (2), eye complaint (1), "tiriça" (severe fever with sore throat) (1)	bark	native	gathered	infusion	oral, bath
Theobroma grandiflorum (Willd. ex Spreng.) K.Schum.	MG_Etn_00696	cupuaçú	tree	diarrhoea (2), belly ache (1), menstrual pain (1)	bark, inner bark	native	cult., gat.	infusion, jiuce with a coffe	oral
**Marantaceae**									
Ischnosiphon gracilis (Rudge) Körn.	MG_Etn_00697	-	herb	snakebites (1)	leaves	native	gathered	infusion	bath

Family/species	Voucher	Vernacular names	Growth form	Traditional uses (citation number)	Plant part used	Origin	Source	Preparation method	Apllication route
**Meliaceae**									
Carapa guianensis Aubl.	MG239791	andiroba	tree	scabies (11), wounds (1), sore throat (2), diabetes (1), headache (1), unblocking milk ducts (1), lice (1)	oill, bark	native	gathered	*in natura*, decoction	topical, oral, bath
Cedrelinga cateniformis (Ducke) Ducke	MG_Etn_00 698	Cedro	tree	worms (2)	bark	native	gathered	decoction	oral
**Moraceae**									
Brosimum parinarioides Ducke	MG_Etn_00 699	amapá-doce	tree	weakness (1)	latex	native	gathered	*in natura*	oral
Helicostylis tomentosa (Poepp. & Endl.) Rusby	MG_Etn_00 700	mururé	tree	diarrhoea (2), wounds (1), painful urination (1)	inner bark, leaves	native	gathered	decoction, juice	oral
**Myristicaceae**									
Virola surinamensis (Rol. ex Rottb.) Warb.	MG239805	virola, bucubeira	tree	diarrhoea (4), toothache (1), earache (3)	latex fruit, bark, inner bark	native	gathered	*in natura*	oral, topical
**Myrtaceae**									
Psidium guajava L.	MG_Etn_00 701	goiaba	tree	diarrhoea (6), belly ache (4), scabies (5)	leaves, bark,	native	cultivated	decoction	bath, oral

Family/species	Voucher	Vernacular names	Growth form	Traditional uses (citation number)	Plant part used	Origin	Source	Preparation method	Apllication route
					inner bark				
Syzygium cumini (L.) Skeels	MG239806	ameixeira	tree	diarrhoea (6), wounds (2)	bark, inner bark	exotic	cultivated	juice, decoction	oral, topical
**Musaceae**									
Musa paradisiaca L.		bananeira	herb	toothache (3), wounds (2), "sapinho" (1), bleeding (1),	resin, latex	exotic	cultivated	*in natura*	oral, topical
**Nymphaeaceae**									
Nymphea sp.		água-pé	herb	erysipela (6), leg pain (1), worms (1)	leaves	-	gathered	cataplasm, decoction	topical, oral
**Olacaceae**									
Ptychopetalum olacoides Benth.	MG_Etn_00657	marapuama	tree	male impotence (3), weakness (1), back pain (1)	root	native	gathered	maceration	oral
**Passifloraceae**									
Passiflora edulis Sims	MG241236	maracujá	climber	feeling anxious (5)	fruit, leaves	native	cultivated	juice, decoction	oral
**Plantaginaceae**									

Family/species	Voucher	Vernacular names	Growth form	Traditional uses (citation number)	Plant part used	Origin	Source	Preparation method	Apllication route
Scoparia dulcis L.	MG239807	vassourinha	herb	prickly heat (8)	whole plant	native	cult., gat.	infusion	bath
**Piperaceae**									
Piper sp.		pimenta malagueta	herb	skin yeast infections (1)	leaves	exotic	cultivated	juice	topical
Piper nigrum L..		pimenta do reino	herb	iInfluenza (2), cough (1)	fruit	exotic	cultivated	syrup	oral
**Phyllanthaceae**									
Phyllanthus niruri L	MG239808	quebra-pedra	herb	urinary calculus (2), urinary infection (2), painful urination (1), kidney problems (5)	root, leaves	native	gathered	decoction	oral
**Phytolaccaceae**									
Petiveria alliacea L.	MG241231	mucura-caá, caatinga da mulata	shrubby	rheumatism (1), joint pain (1), bone pain (1), toothache (2), headache (3), canseira, ear pain (3)	leaves, root	exotic	cultivated	juice, mixture the root with limão resin and tabaco, maceration in acohol, decoction	topical, oral, bath
**Plantaginaceae**									

Family/species	Voucher	Vernacular names	Growth form	Traditional uses (citation number)	Plant part used	Origin	Source	Preparation method	Apllication route
Bacopa monnierioides (Cham.) B.L.Rob.		peixinho	herb	influenza (6), fever (1), headeach (1), stubbornness (3), stomachache (1)	whole plant, leaves	native	gathered	infusion	bath
Conobea scoparioides (Cham. & Schltdl.) Benth.	MG241234	pataqueira	herb	influenza (4), headeach (2), quebrante (1)	whole plant	native	gathered	infusion	bath
**Poaceae**									
Cymbopogon citratus (DC.) Stapf	MG_Etn_00 702	capim-marinho	herb	hoarse throat/voice (1), painful urination (3) , belly ache (3)	leaves	exotic	cultivated	decoction	oral
Coix lacryma-jobi L.	MG239776	lágrimas-de-nossa senhora/ milagre-de-nossa senhora	herb	prostate syntom (1) tuberculosis (1)	leaves	exotic	cultivated	maceration (mixture with canafixe, jucá and water)	oral
Zea mays L.		milho-roxo	herb	measles (3)	fruit	exotic	cultivated	decoction	oral
**Portulacaceae**									
Portulaca pilosa L.	MG_Etn_00 703	amor-crescido	herb	wounds (3)	whole plant	native	cult., gat.	juice	topical
**Rubiaceae**									

Family/species	Voucher	Vernacular names	Growth form	Traditional uses (citation number)	Plant part used	Origin	Source	Preparati on method	Apllication route
Faramea anisocalyx Poepp.	MG_Etn_00 664	corezeiro	tree	phlegm abnormal (1)	bark	native	cult., gat.	juice	oral
Morinda citrifolia L	MG239796	noni	tree	stomachache (1), urinary infection (1)	fruit, leaves	exotic	cultivated	decoction	oral
Uncaria guianensis (Aubl.) J.F.Gmel.	MG_Etn_00 634	unha-de-gato	climber	woman's inflammation (3), bone pain (2)	bark	native	gathered	maceration in alcohol	oral, topical
**Rutaceae**									
Citrus × aurantium f. *aurantium*	MG_Etn_00 704.	laranja-da-terra, laranja	tree	influenza (6), headeach (5), palpitation (4),	leaves	exotic	cultivated	Infuision, decoction, maceration	oral, bath
Citrus limon (L.) Osbeck	MG_Etn_00 705	limão	tree	influenza (21), phlegm abnormal (1), headeache (1), fever (3), dengue (2), hypertension (1), stomachache (1), curuba (1), hiccup (1), dandruff (1)	fruit, seed, leaves	exotic	cultivated	decoction, infusion, syrup	oral, bath
Citrus medica L.	MG_Etn_00 706	limão-cidra	tree	lice (1), hair dandruff (1)	fruit	exotic	cultivated	juice	bath
Coffea arabica L.	MG241239	café	tree	dizziness (1), headeach (1)	seed	exotic	cultivated	infusion	oral

Family/species	Voucher	Vernacular names	Growth form	Traditional uses (citation number)	Plant part used	Origin	Source	Preparation method	Apllication route
Ruta graveolens L.		arruda	herb	headeach (4), toothache (1), quebrante (1), unruly child (1)	whole plant	exotic	cultivated	juice, maceration in alcohol maceration in water with cuia mansa for 24 hours	topical, bath
**Sapindaceae**									
Matayba scrobiculata Radlk.	MG241242	merakati	tree	impingem (1)	stem	native	cult., gat.	cut the stem of the plant, put it in the fire, wait for it to generate a foam and place it on the impingem	Topical
**Sapotaceae**									
Manilkara bidentata subsp. *surinamensis* (Miq.) T.D.Penn.	MG_Etn_00633	maparajuba	tree	belly ache (2)	bark	native	gathered	juice with a coffe	oral
Manilkara elata (Allemão ex Miq.) Monach.	MG_Etn_00641	maçarandub a	tree	diarrhoea (5), belly ache (1)	bark, inner bark	native	gathered	decoction, juice	oral

Family/species	Voucher	Vernacular names	Growth form	Traditional uses (citation number)	Plant part used	Origin	Source	Preparation method	Apllication route
Pradosia cochlearia (Lecomte) T.D.Penn.	MG_Etn_00655	pau-doce	tree	stomachache (1), liver problems (1), irregular menstruation (1)	inner bark	native	gathered	juice	oral
**Simaroubaceae**									
Homalolepis cedron (Planch.) Devecchi & Pirani	MG777791	pau-para-tudo	tree	scabies (3)	bark	native	gathered	maceration	bath
Quassia amara L.	MG239792	quina-quina	tree	malaria (7), scabies (3)	bark	native	cult., gat.	infusion	oral, banth
**Solanaceae**									
Capsicum frutescens L.		pimenta malagueta	herb	skin yeast infections (1)	leaves	exotic	cultivated	juice	topical
Solanum paniculatum L.	MG239802	jurubeba	shrubby	malaria (3)	root	native	cult., gat.	decoction	oral
Physalis angulata L.	MG239803	camapú	shrubby	malaria (2)	root	exotic	cultivated	decoction	oral
**Verbenaceae**									
Stachytarpheta cayennensis (Rich.) Vahl	MG239786	rinxão	herb	inflammation in the uterus (1)	leaves	native	cult., gat.	decoction	oral
Lippia alba (Mill.) N.E.Br. ex Britton & P.Wilson	MG241228	erva-cidreira	herb	pricky heat (3)	leaves	native	cultivated	decoction	oral, bath
**Vitaceae**									
Cissus verticillata (L.) Nicolson & C.E.Jarvis	MG_Etn_00708	cipó-pucá	climber	stroke (4)	leaves	native	gathered	decoction with a	oral

Family/species	Voucher	Vernacular names	Growth form	Traditional uses (citation number)	Plant part used	Origin	Source	Preparation method	Apllication route
								alligator fat	
**Xanthorrhoeaceae**									
Aloe vera (L.) Burm. F		babosa	herb	erysipela (6), burn (3), swellings (3), boil (1), pneumonia (2), influenza (1), cough (1), phlegm abnormal (1), stomachache (1),	leaves	exotic	cultivated	Roasted, syrup from the mixture with the bark or seed of the jucá, sucuúba bark, ginger, and honey. put two days in the sun and two in the dew; decoction	topical, oral
**Zingiberaceae**									
Zingiber officinale Roscoe		gengibre	Herb	influenza (3), cough (3), pneumonia (1), phlegm abnormal (1), stomachache (2), liver problems (1), bone pain (2)	root	exotic	cultivated	syrup; maceration in alcohol; (3) decoction	oral, topical
**Urticaceae**									

Family/species	Voucher	Vernacular names	Growth form	Traditional uses (citation number)	Plant part used	Origin	Source	Preparation method	Apllication route
Cecropia obtusa Trécul/	MG_Etn_00646	imbaúba	Tree	stomachache (1)	root	native	cult., gat.	decoction	oral
Pilea microphylla (L.) Liebm.	MG239797	cama-de-menino-deus	herb	excessive menstruation (1), bleeding (1), inflammation (1)	whole plant	exotic	cultivated	decoction	oral
**Not determinate**									
		cipó-cravo	tree	erysipela (6), leg pain (1), worms (1)	bark		cultivated	infusion	oral
		castanhola	tree		leaves		gathered	decoction	oral
		louro cominho	tree	inflammation (1)	inner bark		gathered	juice	oral
		cipó-diwira	climber	headeach (1), joint pain	bark		gathered	maceration in alcohol	topical
		bucho-de-viado		snakebites (1)	inner bark		gathered	juice	oral, topical
		rosa-branca	herb	bleeding (1)	root	-	cultivated	decoction	oral
